# Assisted Reproductive Technology affects developmental kinetics, *H19 *Imprinting Control Region methylation and *H19 *gene expression in individual mouse embryos

**DOI:** 10.1186/1471-213X-7-116

**Published:** 2007-10-18

**Authors:** Patricia Fauque, Pierre Jouannet, Corinne Lesaffre, Marie-Anne Ripoche, Luisa Dandolo, Daniel Vaiman, Hélène Jammes

**Affiliations:** 1 Biologie de la Reproduction, Hôpital Cochin, AP-HP, Université Paris Descartes, Paris, France; 2 Génétique et Développement, INSERM Institut Cochin U567, CNRS (UMR 8104), Université Paris Descartes, Paris, France; 3 Genetics Department, INRA, Jouy en Josas, France; 4 PHASE Department, INRA, Jouy en Josas, France

## Abstract

**Background:**

In the last few years, an increase in imprinting anomalies has been reported in children born from Assisted Reproductive Technology (ART). Various clinical and experimental studies also suggest alterations of embryo development after ART. Therefore, there is a need for studying early epigenetic anomalies which could result from ART manipulations, especially on single embryos. In this study, we evaluated the impact of superovulation, *in vitro *fertilization (IVF) and embryo culture conditions on proper genomic imprinting and blastocyst development in single mouse embryos.

In this study, different experimental groups were established to obtain embryos from superovulated and non-superovulated females, either from *in vivo *or *in vitro *fertilized oocytes, themselves grown *in vitro *or not. The embryos were cultured either in M16 medium or in G1.2/G2.2 sequential medium. The methylation status of *H19 *Imprinting Control Region (ICR) and *H19 *promoter was assessed, as well as the gene expression level of *H19*, in individual blastocysts. In parallel, we have evaluated embryo cleavage kinetics and recorded morphological data.

**Results:**

We show that:

1. The culture medium influences early embryo development with faster cleavage kinetics for culture in G1.2/G2.2 medium compared to M16 medium.

2. Epigenetic alterations of the *H19 *ICR and *H19 *PP are influenced by the fertilization method since methylation anomalies were observed only in the *in vitro *fertilized subgroup, however to different degrees according to the culture medium.

3. Superovulation clearly disrupted *H19 *gene expression in individual blastocysts. Moreover, when embryos were cultured *in vitro *after either *in vivo *or *in vitro *fertilization, the percentage of blastocysts which expressed *H19 *was higher in G1.2/G2.2 medium compared to M16.

**Conclusion:**

Compared to previous reports utilizing pools of embryos, our study enables us to emphasize a high individual variability of blastocysts in the *H19 *ICR and *H19 *promoter methylation and *H19 *gene expression, with a striking effect of each manipulation associated to ART practices. Our results suggest that *H19 *could be used as a sensor of the epigenetic disturbance of the utilized techniques.

## Background

Many studies have focused on the effects of Assisted Reproductive Technology (ART) on children's health. ART babies now account for approximately 2.2% and 0.6% of all births in France and in the United States, respectively [1]. Singleton pregnancies obtained after ART are at a higher risk for adverse perinatal outcomes than natural pregnancies. These risks include perinatal mortality, preterm delivery, and low birth weight [2-4]. Since low birth weight has been associated to increased rates of cardiovascular and metabolic diseases in adulthood, ART is at least a partial cause of such long-term consequences [5]. Different studies have also reported rare congenital malformations [6-8], chromosomal abnormalities [9], and alterations of cognitive and motor development [10,11]. However it has been difficult to assess the real interaction effects between the mode of conception and the incidence of these anomalies. Recently, the concept of epigenetic risk has associated disturbances of embryonic development with aberrant genomic imprinting. Some reports suggest that ART increases the risk of diseases such as the Beckwith-Wiedemann syndrome (BWS), Prader Willi syndrome (PWS), Angelman syndrome (AS) and Silver-Russel syndrome (SRS) [12-18].

Genomic imprinting leads to a parent-of-origin specific gene expression. Imprinted genes are known to play important roles in regulating embryonic growth, placental functions [19], postnatal metabolic pathways and behavior associated with the control of resources [20]. Moreover, oncogenesis may also be associated with altered epigenetic regulations [21].

Imprinted genes [22] are generally located in clusters, epigenetically marked by DNA methylation on key regulatory sequences (Differentially Methylated Regions, DMRs), by histone modifications (acetylation/deacetylation and methylation) and often associated with antisense RNAs [23,24]. The allele specific methylation of DMRs occurs in germ cells and provides a heritable "memory" that must be maintained throughout fertilization and embryo development. The differential methylation at DMRs is preserved during preimplantation development, in spite of genome-wide changes in global DNA methylation occurring at these early stages [25]. During this period of dynamic epigenetic changes, environmental manipulations, such as hormone-induced superovulation, *in vitro *fertilization (IVF) and embryo culture, could modify genomic imprints and have deleterious effects on later fetal and postnatal stages.

Few studies have reported on the imprinted gene expression in human preimplantation embryos, due to major limitations, such as the scarcity of embryos available for research and the associated ethical restrictions. A monoallelic paternal expression has been shown for both *SNRPN *[26] and *IGF2 *[27,28]. More recently, DNA methylation analysis of control regions of the *SNRPN *gene and *DLK1*/*GTL2 *locus on human early embryo has been reported [29,30]. Most studies made use of mouse models in order to evaluate the impact of *in vitro *fertilization and/or embryo culture using various culture media. Using preimplantation embryos (from the two cell- to the blastocyst stages), an aberrant imprinting of *H19 *gene has been previously found under different culture conditions [31,32]. After implantation, embryonic tissues preserved correct genomic imprints although aberrant *H19 *imprinting was maintained in some placentae [33]. However, alteration of allele-specific methylation of *H19 *gene was shown in fetuses obtained from embryos cultured in medium supplemented with fetal calf serum [34]. Finally studies on the long-term effects of *in vitro *culture on mouse embryos have shown by analyzing development and behavioral parameters in relation to imprinting, that the postnatal development could be affected by embryo culture during the preimplantation period [35,36].

Visual observation of blastocysts suggests a great individual variability of development after IVF and embryo culture. Knowledge about the molecular grounds of such variation is still scarce, but could be of interest for optimizing culture and IVF conditions. Many important questions remain unanswered, such as whether all blastocysts or only a subset lose genomic imprinting and whether a relationship between blastocyst development and genomic imprinting can be observed.

**Figure 1:**
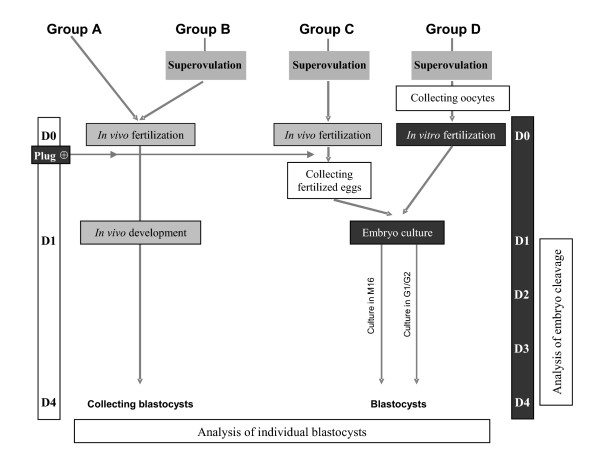
**Experimental group design**. In the two first experimental groups (groups A and B), the fertilization and early embryo development were conducted *in vivo*. Fertilization was realized *in vivo *for group C and *in vitro *for group D. In these two groups (groups C and D), the early embryo development was obtained by culture in two different culture media: M16 and G1.2/G2.2. For *in vivo *fertilization groups (A, B and C), the mating performance was checked by the presence of a vaginal plug. The superovulation of females was induced in groups B, C and D.

**Figure 2:**
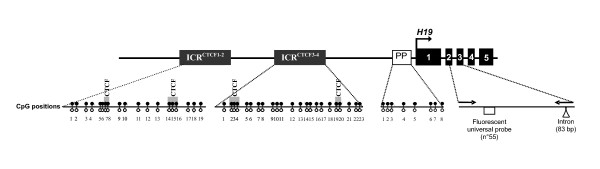
**Schematic representation of the *H19 *gene**. The position of *H19 *ICR, number of CpG nucleotides analyzed for methylation status and position of primer set used for expression analysis are indicated. For methylation analysis of *H19 *ICR, two regions were studied: ICR^CTCF 1–2 ^(574 bp) and ICR^CTCF 3–4 ^(660 bp). The positions of the CpGs analyzed in each sequence are shown by *lollipops *(19 CpGs in ICR^CTCF 1–2 ^and 23 CpGs in ICR^CTCF 3–4^, respectively). The four CTCF binding sites are depicted as grey boxes within the ICR^CTCF 1–2 ^and ICR^CTCF 3–4^. For proximal part of *H19 *promoter (white box with PP), 8 CpGs were analyzed. For expression analysis, real time quantitative RT-PCR was performed using the Taqman technology with two primers (black arrows) chosen to encompass an intron (wedge indicates the exon-exon splice junctions) and a universal probe (white box).

In this study, we wished to address the impact of superovulation, fertilization methods and culture media in the same experimental design (Figure 1). Daily observations of each embryo were carried out in order to establish individual cleavage kinetics and analyze the resulting blastocyst morphology. The maternally expressed *H19 *gene appears to be more sensitive to environmental manipulations than other imprinted genes [31-33,37,38]. To add to the previous knowledge about this locus, we developed techniques enabling to assess the methylation level of both *H19 *Imprinting Control Region (ICR) and proximal part of *H19 *promoter (PP), as well as *H19 *expression level from single embryos brought to the blastocyst stage (Figure 2).

The murine *H19 *gene, located on mouse chromosome 7 distal part, is composed of five exons and four small introns and encodes a 2.3-kb non coding RNA (Figure 2). It is controlled by an ICR located 2 kb upstream of the start of *H19 *transcription and containing CpG dinucleotides methylated on the paternal allele only. Differential methylation of this sequence occurs during male gametogenesis and is maintained during the genome-wide demethylation that occurs before implantation [39-43]. Moreover, on four sites of the unmethylated maternal ICR, the binding of a zinc finger protein called CTCF [44,45] creates a boundary element controlling expression of the neighboring *Igf2 *gene [46-49]. The maternal ICR is also protected from *de novo *methylation which occurs at the blastocyst stage [50]. The proximal promoter is methylated on the paternal allele and contributes to silence *H19 *gene expression [40,51].

In this study, we show that there is an important variability of all analyzed parameters between individual blastocysts within the same experimental group. Nevertheless, environmental manipulations have a high impact on early embryo development, methylation status of *H19 *regulatory regions, and expression of *H19 *gene. The culture conditions strongly influence cleavage kinetics and embryo morphology. The fertilization step followed by embryo culture appears to be a key period for epigenetic changes. In addition, we demonstrate that superovulation is involved in disruption of *H19 *gene expression in blastocysts.

## Results

### *In vitro *development and culture media influence blastocyst maturity

**Figure 3:**
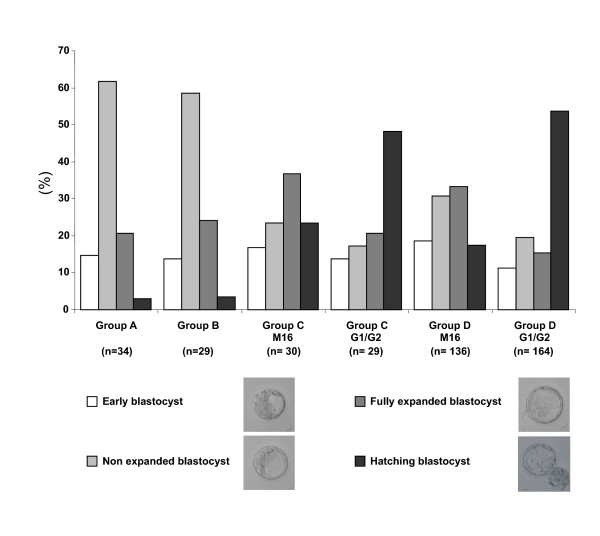
**Blastocyst maturity**. At day 4, each blastocyst was observed and classified into four different categories according to the maturity characteristics (size of embryonic cavity and degree of expansion). The results were expressed as a percentage of the total blastocyst number. In the same experimental group (C or D), significant differences of maturity degree were observed according to the culture medium (χ^2 ^test, P < 0.05)

In order to determine the effect of different environmental manipulations, we analyzed the morphology of blastocysts at day 4 after fertilization (Figure 3). When fertilization and embryo development occurred in vivo, there was no difference of blastocyst maturity between superovulated and non-superovulated mice (groups A and B). After in vivo fertilization and in vitro development (group C), blastocysts were more mature on day 4 than blastocysts which developed in vivo (groups A + B). The proportion of fully expanded and hatching blastocysts was 64.4% in group C versus 25.4% in groups A + B (P < 0.001, χ^2 ^test). The fertilization method did not influence the in vitro embryo development since the percentage of fully expanded and hatching blastocyst was 60.7% after IVF (group D). The drastic effects of in vitro development on blastocyst maturity were modulated by the culture medium used. There was a greater proportion of hatching blastocysts when embryos from groups C and D were cultured in G1.2/G2.2 medium (48.3% and 53.6%) compared to M16 medium (23.3% and 17.3%; P < 0.05, χ^2 ^test). Furthermore, the morphometric analysis at day 4 showed equal blastocyst perimeter and area means in groups A and B. The perimeter and area means tended to increase in groups C and D when blastocysts were cultured in G1.2/G2.2 medium as compared to those in M16 medium. Blastocysts from group C were significantly larger than blastocysts from group B (p < 0.05, t test- data not shown).

### Culture media influence embryo cleavage kinetics and have various impacts according to the fertilization method

**Figure 4:**
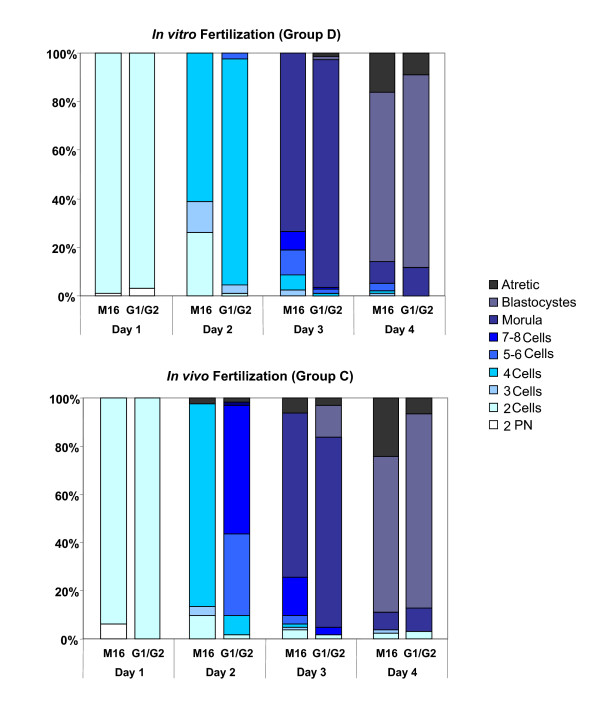
**Embryo cleavage kinetics according to culture media and fertilization method**. Daily observations for all cultured zygotes and color code classification according to the cell number are depicted. At day 0, two pronuclei were classically observed (2 PN) and defined the zygote stage. Zygotes obtained after *in vitro *fertilization (Figure **4A **group D) or after *in vivo *fertilization (Figure **4B **group C) were then cultured in M16 or G1.2/G2.2 medium. The results are expressed as percentage of total zygote (PN) number at day 0 for each experimental group and each culture condition. At each culture day, the number of atretic embryos was determined based upon the observation of necrosis signs.

To analyze the effects of different culture media on preimplantation embryo development, a detailed analysis of cleavage kinetics was done each day for embryos from *in vivo *(group C) and *in vitro *fertilization (group D), cultured in both media from one cell stage to blastocyst stage (Figure 4). Differences of cleavage kinetics between both media were observed as early as day 2 and maintained until day 4 of culture, with a faster cell cycle in the sequential G1.2/G2.2 medium than in M16 medium in both groups C and D.

Almost all zygotes from group D (Figure 4A) developed to the 2 cell-stage at day 1 of culture (99.0% in M16 medium (n = 200 zygotes) and 97.1% in sequential G1.2/G2.2 medium (n = 204 zygotes)). At day 2, difference of cleavage kinetics was observed with 61.0% of embryos at the 4-cell stage in M16 medium versus 93.1% in G1.2/G2.2 medium (P < 0.05, χ^2 ^test). This difference of cleavage kinetics between both media was maintained until day 4 of culture. At day 3 and day 4, a significantly higher number of embryos reached the morula and blastocyst stage in G1.2/G2.2 compared to M16 medium (95.1% versus 72.5% at morula stage and 79.4% versus 70.0% at blastocyst stage respectively; P < 0.05, χ^2 ^test). Finally the number of atretic embryos was two fold higher in M16 compared to G1.2/G2.2 medium at day 4 (16% versus 8.8%; P < 0.05, χ^2 ^test).

Zygotes from group C (62 in G1.2/G2.2 and 82 in M16) exhibited similar differences in cleavage kinetics between the two media. Embryo cleavage occurred faster in the sequential G1.2/G2.2 compared to M16 medium (Figure 4B). We also found in group C that the percentage of atretic embryos at day 4 was significantly increased after culture in M16 compared with G1.2/G2.2 medium (24.4% versus 6.5%; P < 0.05, χ^2 ^test). In conclusion, we observed that embryos from group C reached the second cell cycle earlier than those from group D with a significant enhancing effect of G1.2/G2.2 sequential medium.

### Methylation analysis

**Figure 5:**
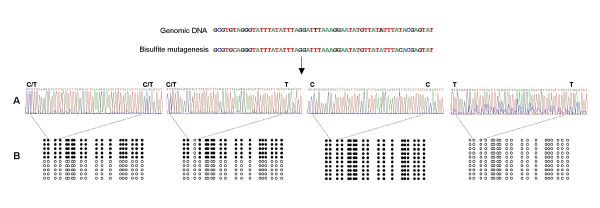
**Methylation analysis of individual blastocysts by bisulfite conversion followed by direct sequencing and cloning/sequencing**. For each analyzed blastocyst, bisulfite mutagenesis was performed. After PCR amplification the methylation status of CpG positions was determined by direct sequencing (Figure **5A**) and cloning/sequencing (Figure **5B**). When Single Nucleotide Polymorphism (C/T) was observed by direct sequencing, the proportion of C in the clone sequences was approximately 50%. When only C or T were detected by direct sequencing, all sequences of analyzed clones presented a methylated or an unmethylated status respectively. Reading the direct sequences, for each blastocyst, the presence of C/T, C or T at one CpG position is represented by the black (methylated) and white (unmethylated) lollipops (Figure **5C**).

**Figure 6:**
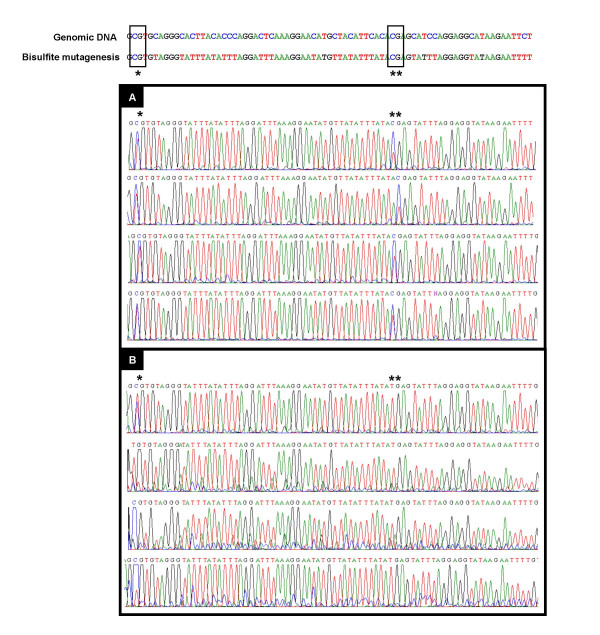
**Direct sequencing analysis of *H19 *ICR in individual blastocysts**. Eight examples of sequences obtained by direct sequencing of bisulfite mutated genomic DNA of individual blastocysts without (Figure 6A) or with methylation defects (Figure 6B) are shown.

The methylation status was determined by two methods: cloning and sequencing of PCR products from amplification of bisulfite mutated genomic DNA of individual blastocysts and direct sequencing of this same PCR product. To validate the direct sequencing approach, ten clones from each blastocyst were sequenced as well as the PCR product, in both orientations. Overall, one hundred clones were analyzed for a total of ten blastocysts from the different experimental groups (Figure 5). For each CpG position analyzed, the allele specific methylation status observed by cloning-sequencing analysis appeared always as a single nucleotide polymorphism (C/T), also visible after direct sequencing (Figure 5). Alternatively, the unmethylated status of a given CpG obtained by cloning and sequencing appeared as a thymine by direct sequencing. By contrast, a methylated status observed by cloning/sequencing appears as a cytosine by direct sequencing. Therefore, we concluded that the direct sequencing analysis was clearly representative of the blastocyst methylation status. The expected allele-specific methylation status was easily discriminated from absence of methylation as shown in Figure 6. After this validation step, direct sequencing was systematically used in further experiments.

### Fertilization method followed by embryo culture affects the methylation status of the *H19 *ICR and proximal part of *H19 *promoter with modulation by the culture medium

**Figure 7:**
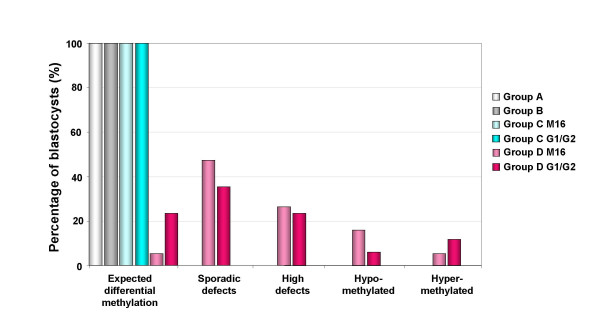
**Methylation status of *H19 *ICR (A: ICR^CTCF 1–2 ^region analysis and B: ICR^CTCF 3–4 ^region analysis) for individual blastocysts determined by bisulfite/sequencing analysis**. For each group, methylation status of individual blastocysts was analyzed by direct sequencing and is represented by the black and white lollipops. The CTCF binding sites are shaded in grey. Only examples are shown for clarity.

The methylation status of the *H19 *ICR^CTCF 1–2 ^region was assessed *H19 *for 19 CpG positions on 85 individual blastocysts. A preserved differential methylation of *H19 *ICR^CTCF 1–2 ^was found for each analyzed CpG of embryos fertilized *in vivo *as measured in 8 blastocysts of group A, 26 of group B, 8 of group C cultured in M16 medium and 7 of group C in G1.2/G2.2 medium (Figure 7). Methylation defects were often observed after *in vitro *fertilization in blastocysts of group D and in both culture media (n = 36). In this group, the proportion of individual blastocysts with unmethylated alleles and the number of non-methylated CpGs were more important when cultured in M16 compared to sequential G1.2/G2.2 medium (18 out of 19 in M16 and 13 out of 17 in G1.2/G2.2 medium – Figure 7). Only one blastocyst out of 19 from group D M16 and 4 out of 17 from group D G1.2/G2.2 had a correct differential methylation status at all the CpG positions. The unmethylated CpGs appeared preferentially outside of the CTCF binding sites, thereby possibly reducing the deleterious consequences of the anomaly.

Analysis of 23 CpG positions of the *H19 *ICR^CTCF 3–4 ^region was done in 27 individual blastocysts: 10 of group B, 13 of group D cultured in M16 and 4 of group D cultured in G1.2/G2.2. Methylation defects of the *H19 *ICR^CTCF 3–4 ^region were also observed in blastocysts of group D obtained from both culture media but in a lower number of blastocysts as compared to the *H19 *ICR^CTCF 1–2 ^region (Figure 7).

**Figure 8:**
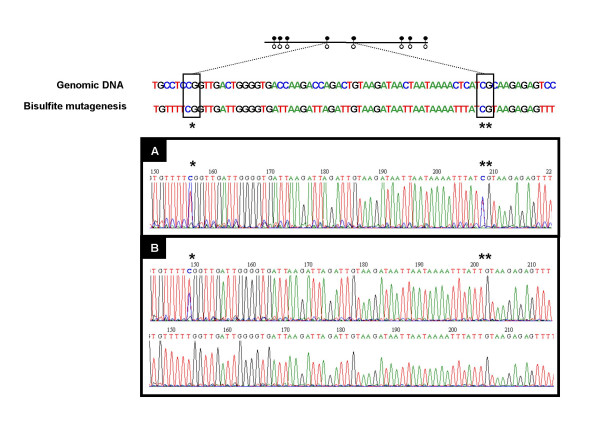
**Direct sequencing analysis of proximal part of *H19 *promoter in individual blastocysts**. Examples of sequences obtained by direct sequencing of bisulfite mutated genomic DNA of *H19 *promoter in individual blastocysts without (Figure **8A**) or with methylation defects (Figure **8B**) are shown.

In order to identify possible epigenetic defects affecting the *H19 *promoter, normally differentially methylated, we also assessed its methylation status for 8 CpG positions (Figure 8) on 17 individual blastocysts. For blastocysts fertilized and developed *in vivo*, the expected allele specific methylation was found (6 individual blastocysts). Methylation defects were observed for 5 blastocysts out of 6 from group D M16 and 4 out of 5 from group D G1.2/G2.2.

**Figure 9:**
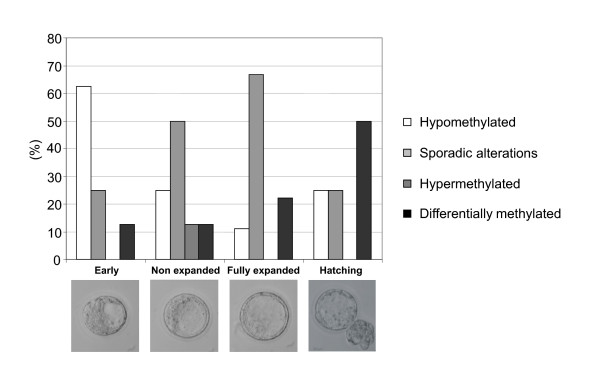
**Methylation status of ICR^CTCF 1–2 ^according to the blastocyst maturity in group D**. For each maturity stage, the results are expressed as a percentage of the blastocyst number.

Finally, the methylation status was analyzed according to the maturity of blastocysts obtained from group D (Figure 9). Clearly, the expected specific allele methylation was more often observed in hatching blastocysts, which correlates methylation defects with abnormal or failing developmental processes.

### Superovulation disturbs *H19 *gene expression in individual blastocysts

**Figure 10:**
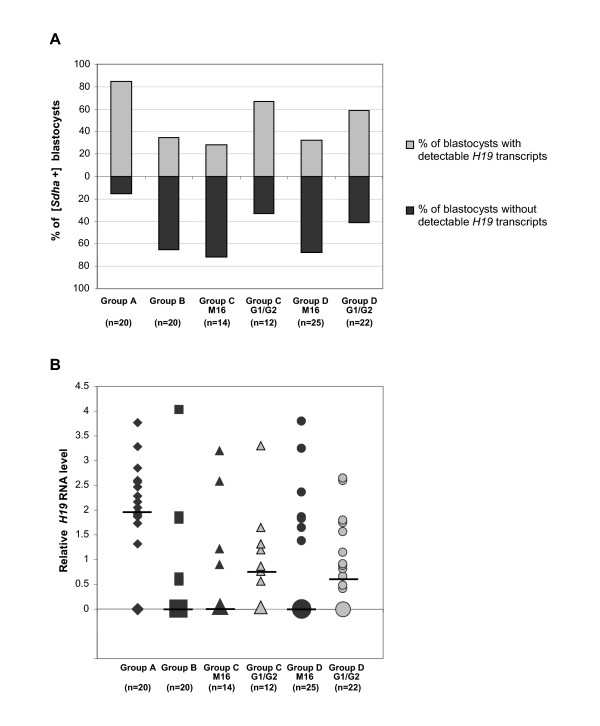
***H19 *gene expression patterns in individual blastocysts according to experimental groups**. *H19 *expression was determined by real time quantitative RT-PCR and calculated taking into account expression of a housekeeping gene (*Sdha*) and an internal standard sample. The results are presented as proportion of blastocysts with or without detectable *H19 *transcripts in *Sdha *expressing blastocysts (Figure **10A**). The relative *H19 *RNA level is expressed according to the equation: *H19 *RNA quantity = 2 ^-ΔΔCt ^with ΔΔCt = (Ct(*H19*)-Ct(*Sdha*)_sample _- Ct(*H19*)-Ct(*Sdha*)_standard_). For each group, individual blastocyst expression is represented. The bars indicate the median expression value. The size of symbols is proportional to blastocyst number with the same *H19 *expression (Figure **10B**).

Analysis of *H19 *gene expression by real time quantitative RT-PCR was done in 123 individual blastocysts: 21 from group A, 21 from group B, 16 from group C M16, 12 from group C G1.2/G2.2, 26 from group D M16 and 26 from group D G1.2/G2.2. Expression of the *Sdha *housekeeping gene was detected in 113/123 blastocysts (90.8% *Sdha*+ blastocysts). The proportion of blastocysts without *Sdha *detection was similar in all groups, suggesting that the quality of mRNA preparation was not influenced by the different modes of embryo collection. Among the *Sdha+ *blastocysts, two subpopulations were observed, one with detectable *H19 *transcripts (*H19+*) and the other without detectable *H19 *transcripts(*H19-*). The proportion of *H19+/H19- *blastocysts varied according to the experimental groups (Figure 10A). The proportion of *H19+ *blastocysts was significantly higher in group A versus each other group (*P *< 0.01, χ^2 ^test). In group C, this proportion was higher for embryos cultured in G1.2/G2.2 medium versus M16 medium (*P *< 0.05, χ^2 ^test). In group D, the same difference was visible, although not significant. The calculated mean of relative *H19 *RNA expression level taking into account the housekeeping gene (*Sdha*) expression as an internal standard sample, showed no significant difference between the groups, probably owing to the high variability of *H19 *expression inside each group (*P *> 0.05, t-test; Figure 10B). However, the median value in group A was higher than that of groups where fertilization occurred after superovulation, as shown by a non-parametric test (Mann and Whitney rank test: *P *< 0.0001).

In all experimental groups analyzed, the *H19 *expression potentiality of blastocysts was not correlated with the maturity stage. To further investigate the potential importance of *H19 *gene expression variability, we also analyzed the cleavage kinetics of embryos from *H19 *null mutated mice (*H19Δ3*) [52]. The oocytes lacking *H19 *produced from *H19Δ3 *mice were submitted to *in vivo *or *in vitro *fertilization procedures, followed by embryo culture in G1.2/G2.2 medium (Groups C^*H19Δ3 *^and D^*H19Δ3*^). Similar fertilization rate, early embryo development and blastocyst maturity were observed in this mouse model as compared to the previously analyzed groups C and D (data not shown). We conclude from these results that lack of *H19 *does not affect these first preimplantation stages.

## Discussion

The results described in our study highlight the interest to investigate precisely the impact of ART micromanipulations on changing epigenetic patterns. We combined several factors of ART process such as superovulation, IVF and culture medium and analyzed their putative effects on early embryo development. Furthermore, we performed for the first time on individual blastocysts, the methylation and expression analysis of the imprinted *H19 *gene, as a sensitive marker to environmental alterations. We clearly show that there is an important variability between individual blastocysts, that each environmental manipulation has an impact on embryo development and that the choice of the culture medium can modulate embryo responsiveness.

In our study, *in vitro *fertilization was routinely performed in M16 medium. Using the observation of two pronuclei as a criterion of optimal fertilization, a high rate of zygotes was obtained (88.7%). The same medium was also used for *in vitro *blastocyst development. This basic culture medium was then compared with the more complex G1.2/G2.2 sequential medium which is commonly utilized in human IVF. Using these two culture systems, our results showed significant differences in the kinetics of embryo cleavage and embryo morphology up to the blastocyst stage.

The embryo cleavage kinetic differences were observed as early as the second cell division at day 2. The presence of non essential amino acids in G1.2 medium (alanine, asparagine, aspartate, glutamate, glycine, alanyl-glutamyl, proline, serine) compared to M16 medium, improved the development to the 4-cell stage as previously shown [53]. The addition of essential amino acids in G2.2 medium from day 2 of culture is known to increase the frequency of hatching blastocysts, in association with a significant increase of cell number, preferentially in the inner cell mass of embryos [54-56]. The uptake of specific amino acids, notably leucine, isoleucine and valine, has been shown to occur during the blastocyst formation and expansion *in vitro*, suggesting a critical developmental role [57].

Our data also show that the fertilization method modified the sensitivity of embryos to culture medium. After *in vivo *fertilization, the modifications of cleavage kinetics induced by the G1.2/G2.2 medium were more marked than after *in vitro *fertilization. At day 2, the majority of embryos cultured in M16 reached the 4-cell stage while 50% of embryos cultured in G1.2/G2.2 were already at the 6-8-cell stages. These results highlight the importance of metabolic embryo capacities which are established at the moment of fertilization and implicated in its future potential development [31,33,58].

Interestingly, the changes in embryo cleavage kinetics observed at day 2 occur concomitantly with the zygotic genome activation in mice. The maternal-to-zygotic transition of gene expression is triggered by the degradation of oocyte-specific transcripts followed by zygotic gene activation. This process initiates during the 1-cell stage and is evident by the 2-cell stage in mouse [59-61]. The embryo cleavage kinetics and the blastocyst morphology could be a direct reflection of the appropriate pattern of gene expression. The fertilization method and the medium composition could have a potential impact on this appropriate pattern of gene expression initiated during the one-cell stage and could modify the metabolic capacities of the embryo.

In our study, the *H19 *gene has been chosen for its implication in the regulation of feto-placental growth by way of action on *Igf2 *expression [62-64]. Moreover, a possible link between ART and *H19 *imprinting disorders was suggested in humans [14]. In mouse, *H19 *imprinting also appears more sensitive to *in vitro *manipulations than other imprinted genes[31-34,37,38]. In the present study, the *H19 *ICR methylation status has been analyzed using bisulfite conversion on single blastocyst DNA. Whatever the four groups analyzed, 80% of the blastocysts yielded a PCR product. Therefore, a possible PCR bias due to the small amount of remaining amplifiable DNA after bisulfite treatment cannot explain the methylation defects found exclusively in blastocysts from group D. The expected allele-specific methylation status at four CTCF sites in the *H19 *ICR was observed for all blastocysts obtained from *in vivo *fertilization and development without or with female superovulation (group A and B) and for all blastocysts obtained from *in vivo *fertilization and *in vitro *development (group C). In contrast, in the *in vitro *fertilization group (group D), methylation defects were observed with different degrees according to the culture medium. The proportion of blastocysts presenting epigenetic alterations of the *H19 *ICR was higher in M16 medium as compared to sequential medium. The ICR^CTCF 1–2 ^region showed major methylation defects as compared to ICR^CTCF 3–4 ^where the differential methylation appeared to be maintained. A relation between accurate imprinting marks and developmental ability was suggested by our results since a higher number of hatching blastocysts exhibited a differential methylation.

In a previous study, the methylation of *H19 *ICR region containing CTCF sites 1 and 2 performed on pools of embryos, was affected by the culture in Whitten's medium, while culture in KSOM+AA approximated more closely the *in vivo *situation [33]. In comparison to these data, our study was performed on individual blastocysts and extended the analysis of the ICR to all four CTCF binding sites as well as to the proximal part of *H19 *promoter. This suggests that the ICR^CTCF 3–4 ^region may be relatively preserved from epigenetic alterations after IVF, whereas the ICR^CTCF 1–2 ^is more affected by IVF and culture medium. Nevertheless, the discrepancy between our results and previous studies could be explained by strain-specific modifiers affecting the maintenance of *H19 *imprint as mentioned by Doherty et al. [31-34,37,38]. The authors found that embryos derived from a *Mus musculus castaneus *female and C57BL/6J male displayed a loss of *H19 *imprinting when cultured in Whitten's medium whereas embryos produced by reciprocal mating showed appropriate imprint of the *H19 *gene under identical conditions.

After *in vivo *fertilization, the hemi-methylation of ICRs appears remarkably maintained at the early embryo stage, in spite of genome wide changes in DNA methylation [65]. For *H19 *ICR, the paternal allele is methylated [66] and protected against the genome wide demethylation. Thus, the ICR^CTCF 1–2 ^methylation defects might be due to a partial failure of this protection, possibly related to a modification of paternal chromatin structure. It is poorly understood which protein factors are involved in this process. *In vitro *fertilization leads to notably different environmental conditions compared to the maternal conditions. This could generate deficiencies in uptake of substrates essential for accurate methylation process. It would be interesting to know whether other ICRs are also perturbed by the *in vitro *fertilization step.

The hypo-methylation of ICR^CTCF 1–2 ^and ICR^CTCF 3–4 ^lead us to speculate that a loss-of-imprinting could appear in blastocysts of group D since the methylation of *H19 *gene is reported as crucial for its imprinted expression [31,33,38,39,49]. Finally, we evaluated the global expression level of *H19 *gene by quantitative RT-PCR in individual blastocysts. We did not find any clear association between the abnormal methylation of *H19 *ICR/promoter and a disruption in *H19 *gene expression. However, our results show that superovulation clearly diminishes *H19 *detectable transcripts. This suggests that mature oocytes obtained from superovulation could have cytoplasmic and nuclear maturation defects. Interestingly, *in vitro *embryo development on complex culture medium appeared to improve *H19 *expression levels and to compensate the detrimental effects of superovulation. Results obtained on oocytes from *H19Δ3*mice showed similar results to normal oocytes for groups C and D. Thus, lack of *H19 *does not seam to modify embryo reactivity to ART conditions during preimplantation stages. It remains to be investigated if later embryonic stages can be affected by reduced levels of *H19 *RNA.

Our data suggest that preimplantation embryos could possess an important flexibility and could be capable of compensating suboptimal environmental manipulations as shown by a strong individual embryo variability. These various embryo phenotypes could be involved in efficiency differences in implantation and fetal development.

## Conclusion

In summary, the challenge of our study was to determine whether ART manipulations result in altered methylation status or in altered expression of the *H19 *gene. We demonstrate here that superovulation affects *H19 *RNA expression levels. This supports the hypothesis that oocyte quality and oocyte maturation could have an impact on embryo quality and development. We also show that in *vitro *fertilization disturbs DNA methylation at specific locations inside the *H19-Igf2 *locus. In addition, we demonstrate that embryo culture affects DNA methylation as previously shown but culture medium can limit epigenetic changes. Finally, the culture medium also modulates cleavage kinetics at early stages of development.

Each of these steps can affect expression of *H19 *gene as well as methylation of the regions controlling its imprinted status, in a distinct manner perhaps with no dramatic outcome. *H19 *could be used as a sensor to investigate more deeply each of the parameters in the mouse system in order to improve human experimental procedures.

## Methods

### Animals

Five to six weeks old F1 (C57BL/6/CBA) females and eight to nine weeks old F1 males (Charles River Laboratories, L'Arbresle, France) were maintained in an animal facility at normal temperature (24–26°C) and 14 h light/10 h dark photoperiods with free access to water and food.

Females homozygous for the *H19 *gene deletion (*H19Δ3*) [52] were used to obtain oocytes lacking *H19 *expression. These oocytes were fertilized by sperm from eight to nine weeks old F1 males.

Procedures for handling and experimentation followed ethical guidelines established by the Federation of European Laboratory Animal Science Associations.

### Experimental design

The experimental group design is shown in Figure 1. Mice were divided into four different experimental groups (A, B, C and D). In groups A and B, blastocysts were collected after *in vivo *fertilization and *in vivo *development, without superovulation of the females in group A and with superovulation in group B. In group C, blastocysts were obtained after *in vivo *fertilization and *in vitro *development from one cell to blastocyst stage. All females were superovulated. In group D, blastocysts were obtained after *in vitro *fertilization and *in vitro *development. All females were superovulated.

The embryos from groups C and D were cultured either in M16 medium (Sigma-Aldrich, Lyon, France) (C M16 and D M16 groups) or in sequential G1.2/G2.2 medium (JCD Laboratories, Lyon, France) (C G1.2/G2.2 and D G1.2/G2.2 groups). The compositions of M16 and G 1.2/2.2 are similar to those of Whitten's and KSOM+AA media, respectively.

### Superovulation

Females from groups B, C and D were superovulated by intraperitoneal (i.p.) injection of 8 IU (0.1 ml) Pregnant Mare Serum Gonadotropin (PMSG, Chronogest; Intervet, Beaucouzé, France), followed 47 h later by an i.p. injection of 5 IU (0.1 ml) of human Chorionic Gonadotropin (hCG, Chorulon; Intervet).

### *In vitro *fertilization

All media and culture dishes were equilibrated overnight in the incubator under a humidified atmosphere of 5.5% CO_2 _at 37°C before use. At 13 h post-hCG, cumulus-oocyte complexes were recovered from oviducts in M2 medium (Sigma-Aldrich) supplemented with 7 mg/ml of BSA (Sigma-Aldrich). After rinsing in M16 medium, cumulus-oocyte complexes were kept in the incubator (37°C, 5.5% CO_2 _in air) in 100 μl drops of M16 medium covered with paraffin oil (JCD Laboratories). Sperm was collected from the cauda epididymis and capacitated for 90 min in M16 medium supplemented with 7 mg/ml of BSA at 37°C and 5.5% CO_2_. Sperm insemination was realized 15 h post-hCG at a concentration of 1.10^6 ^sperm/ml. After 5 h at 37°C and 5.5% CO_2_, in order to eliminate cumulus cells and spermatozoa a mechanical decoronisation was performed using tips of 125 μm diameter on a STRIPPER^® ^(JCD, Lyon, France) followed by several washes in M16 medium (see Additional Files 1 and 2). Successfully fertilized eggs were determined by the presence of two pronuclei and transferred to 30 μl drops of fresh medium covered with paraffin oil. To analyze the culture medium effects, two different media were tested: the M16 medium and the sequential G1.2/G2.2 medium containing amino acids. The embryo culture was conducted to blastocyst stage at 37°C and 5.5% CO_2_., The culture medium was changed every day.

### Collecting fertilized eggs

*In vivo *embryos at one cell stage (fertilized eggs at zygote stage) were obtained from F1 females mated individually with F1 males after hCG injection. The following morning, females were checked for successful mating by the presence of a vaginal copulation plug. The zygotes were retrieved by flushing the oviducts with M2 medium supplemented with BSA (7 mg/ml) at day 1 *post-coitum *(p.c.), 21–23 h post-hCG. After mechanical decoronisation and washes, zygotes selected by the presence of two pronuclei were cultured in the two different culture media under similar conditions as described for *in vitro *fertilization.

### Collecting blastocysts

Embryos at the blastocyst stage were obtained from F1 females mated individually with F1 males by flushing the uterus between 3.5 and 4.5 days p.c. with M2 medium supplemented with BSA (7 mg/ml). In order to eradicate cell contamination, several washes were performed using tips adapted on STRIPPER^®^.

### Embryo cleavage assessment and morphology analysis

All zygotes from groups C and D were daily observed under an inverted microscope with Hoffman Modulation Contrast^®^optics (TE2000-S – Nikon). Eight hours post-sperm insemination, evidence of fertilization was assumed by the presence of two pronuclei in normally fertilized zygotes. The kinetics of embryo cleavage was determined by daily observations (from day 1 to day 4) under the same optical conditions (at 400× magnification).

All blastocysts from the four experimental groups were put individually into a single drop (20 μl). For the blastocyst evaluation, the degree of embryonic cavity (blastocoel) and expansion were used to categorize blastocyst maturity. Embryos were classified as early blastocyst (blastocoel of less than half of the embryo volume), non expanded blastocyst (blastocoel that is at least half of the embryo volume), fully expanded (blastocoel volume larger than that of the early embryo, with a thinning zona) and hatching blastocyst (the trophectoderm starting to herniate through the zona). Each blastocyst was identified, photographed and tracked by computer database. All images were taken with ACT-1 software and morphology parameters such as perimeter and area were analyzed with ImageJ software.

### DNA methylation analysis

Analysis of the *H19 *ICR and proximal part of *H19 *promoter methylation status was determined by cloning and sequencing of bisulfite-treated genomic DNA. Methylation of the four CTCF binding sites was assessed (Figure 2). Briefly, after *in vitro *culture or *in vivo *development, blastocysts were collected and individually put into an agarose bead (one blastocyst/20 μl of 2% low melting agarose; LMP agarose ultrapure, Invitrogen, Cergy Pontoise, France).

#### DNA extraction

To prepare genomic DNA, each bead was submitted to proteinase K treatment (0.2 mg/ml, Invitrogen) in lysis buffer (0.5 M EDTA, pH 8.0) overnight at 37°C. Thus, the genomic DNA from only one blastocyst (60 cells, approximately 360 pg of DNA) was trapped into one agarose bead.

#### Bisulfite treatment

Each bead was individually transferred to an eppendorf tube. After washing in sterilized H_2_O, the agarose trapped DNA was denaturated in freshly prepared sodium hydroxide solution (0.2 M NaOH, 15 min, 37°C). An extemporally prepared solution of sodium bisulfite was added (2.8 M sodium bisulfite, 0.5 mM hydroquinone, 0.6 M NaOH, pH 5.0) to each bead. The reaction mixtures were overlaid with mineral oil and incubated at 50°C for 4 h in the dark. The beads were washed in 10 mM Tris-HCl, 10 mM EDTA, pH 8.0 (8 washes, 10 min). To stop the bisulfite conversion, desulfonation was performed by incubation of the beads in 0.2 M NaOH, at 37°C for 15 min (2 times). Finally, the beads were washed one more time, in 10 mM Tris-HCl, 10 mM EDTA, pH 8.0 (8 washes, 10 min), transferred to PCR tubes in 10 μl of sterilized H_2_O and stored at -80°C.

#### PCR amplification of bisulfite-treated DNA

All PCR primers were designed to be fully complementary to the deaminated strand. Figure 2 represents the two ICR regions and proximal promoter region studied with the number of CpGs analyzed and Table 1 lists the accession numbers, nucleotide positions, PCR primers, and sizes of PCR products. After individual melting of each blastocyst-bead, the bisulfite converted DNA was amplified by a nested PCR protocol previously described [40,67]. The PCR program consisted of a denaturing step of 5 min at 95°C followed by 35 cycles of 30 sec. at 95°C, 120 sec. at 47°C or 50°C and 90 sec. at 72°C. The presence of amplified products was analyzed by electrophoresis on 2% agarose gel. The PCR products were purified by Minelute PCR purification kit (Qiagen, Courtaboeuf, France) and finally eluted in 20 μl of RNase/DNase-free water. PCR products were then sequenced or cloned and sequenced using the pGEM-T EasyVector System (Promega, Charbonnières, France). The nucleotide sequences obtained by cloning/sequencing or by direct sequencing of PCR products were analyzed with BioEdit Sequence Alignment Editor and BiQ analyser program [68]. The efficiency of the genomic DNA conversion by bisulfite was 99.9%. After the nested PCR, 80.0% of blastocysts were amplified.

**Table 1:** PCR primers for DNA methylation and expression analysis

Gene	Primer number	Position	Primer sequence (5' to 3')	AT (°C)	Cycle number	PCR (bp)	Sequence reference
DNA methylation of *H19*							
ICR^CTCF 1–2^	Primers 1						AF049091
	Sens		GATTAGATAGTATTGAGTTTGTTTGGAGT				
	AntiSens		ATCAAAAACTAACATAAACCCCT	47	35		
	Primers 2-Nested						
	Sens	1377/	GAGAAAATAGTTATTGTTTATAGTTTT				
	AntiSens	1971	CCTCATTAATCCCATAACTAT	47	35	574	
ICR^CTCF 3–4^	Primers 1						AF049091
	Sens		GGTTTTTTTGGTTATTGAATTTTAAAATTAG				
	AntiSens		AAAAACCATTCCCTAAATACACAAATACC	47	35		
	Primers 2-Nested						
	Sens	2825/	TTAGTGTGGTTTATTATAGGAAGGTATAGAAGT				
	AntiSens	3433	TAAACCTAAAATACTCAAAACTTTATCACAAC	47	35	660	
Proximal promoter	Primers 1						AF049091
	Sens		TGATTGGTTAGTTTTTGAGTTTT				
	AntiSens		TAATAACTAATTTAAACACTCCTCACC	50	40		
	Primers 2						
	Sens	4648/	GGTGTTTTGATTTGTGG				
	AntiSens	5019	TAATAACTAATTTAAACACTCCTCACC	50	40	371	
Expression analysis							
*H19*	Primers						NR_001592.1
	Sens	6995/	ACATGACATGGTCCGGTGT				
	AntiSens	7145	TCCCATGGTGTTAACGAAGGC				
	Universal Probe 55		GGAGAGGA	54	55	69	
*SDHA*	Primers						NM_023281.1
	Sens	1435/	CCCTGAGCATTGCAGAATC				
	AntiSens	1504	TCTTCTCCAGCATTTGCCTTA				
	Universal Probe 80		CCTGGAGA	54	55	70	

### Quantitative RT-PCR analysis of *H19 *expression

#### RNA preparation

Collected blastocysts were visualized under the microscope and individually transferred to PCR tubes in 1 μl of culture media. The total RNA was purified according to the RNeasy Micro technology and following the manufactor instructions (Qiagen), eluted in a final volume of 14 μl and stored at -80°C (1 blastocyst, 60 cells, about 100 pg of total RNA).

#### cDNA production

Using the total RNA preparation (14 μl), RNase-free water, hexamer solution and dT oligonucleotide primers were added to each tube. The mixture was then heated to 65°C for 5 min and quickly chilled on ice. RNase inhibitor (40 IU/μl), 0.1 M DTT, SuperScript II RT (100 IU/μl) and RT-buffer (Invitrogen) were added for cDNA production in a total volume of 25 μl. The reaction was performed in a thermal cycler with the following conditions: room temperature for 10 min, 42°C for 50 min and 70°C for 15 min. The cDNA was stored at -80°C.

#### H19 expression by real time quantitative RT-PCR

5 μl aliquots of cDNA were used for one PCR amplification. For each analyzed gene, the real time quantitative RT-PCR was performed using Taqman technology with the Universal probe library kit (Roche Applied Science, Mannheim, Germany). Briefly, two primers were designed for each gene by the universal probe library program [69], chosen to encompass at least one intron to avoid amplification of genomic DNA and compatible with a universal probe designed by the program (Table 1, see primers and probes used; Figure 2). The LightCycler experimental program for an assay using the LighCycler Taqman Master (Roche Applied Science) usually consisted of a pre-incubation step to activate the Fast start DNA polymerase and denature the DNA for 10 min at 95°C, followed by 55 cycles of 10 sec at 95°C, 10 sec. at primer dependent temperature (Table 1) and 10 sec. at 72°C and finished by a cooling step of 30 sec. at 40°C. Succinate dehydrogenase complex, subunit A (*Sdha*) was used as housekeeping gene. Each primer-probe set was first tested for amplification on mouse placenta cDNA standards. To exclude amplification from genomic DNA, each primer-probe set was tested using two samples, originated from the same blastocyst RNA preparation, previously incubated with reverse transcriptase or without reverse transcriptase. A similar primer efficiency (around 1.89) was observed for the two primer-probe sets used (*H19*-55; *Sdha*-80). *H19 *expression was calculated taking into account an internal standard sample: *H19 *expression = (Ct(*H19*)_sample _- Ct(*SDHA*)_sample_) - (Ct(*H19*)_standard _- Ct(*SDHA*)_standard_).

### Statistics

At least three replicate experiments were done to obtain blastocysts in different groups and sub-groups. The χ^2 ^test was used for the comparison of binary variables and continuous variables were compared using the independent Student's t-test, when appropriate. The significance level was set at 5% (*P *< 0.05). The results of this parametric analysis were confirmed using a non parametric Mann and Whitney rank test.

## Authors' contributions

PF coordinated the study and performed the experimental work for animal work, qRT-PCR, and methylation analysis, PJ conceived the project and was involved in the redaction of the paper, CL and MAR provided technical assistance for animal work, LD participates in redaction of the paper, HJ and DV conceived the project, participate in the experimental designs (methylation analysis and qRT-PCR, respectively) and were involved in the redaction of the paper. All authors have read and approved the final version of the manuscript.

## Supplementary Material

Additional file 1 **Forty blastocysts**. Forty photographs of individual blastocysts. After stringent procedures (mechanical decoronisation and washes), neither granulosa cells nor spermatozoa could be observed. Click here for file

Additional file 2 **Spermatozoa or granulosa cells observation**. No contamination by sperm or granulosa cells could be missed since they are easily observed at the one cell stage. In fact since we consistently used stringent washing and decoronisation procedures, possible events of contamination could always be excluded (see additional file 1). Click here for file
