# Supplementary figures and images for: Assisted Reproductive Technology affects developmental kinetics, H19 Imprinting Control Region methylation and H19 gene expression in individual mouse embryos
Source: BMC Dev Biol. 2007 Oct 18;7:116. doi: 10.1186/1471-213X-7-116 (PMC2169233; doi:10.1186/1471-213X-7-116)

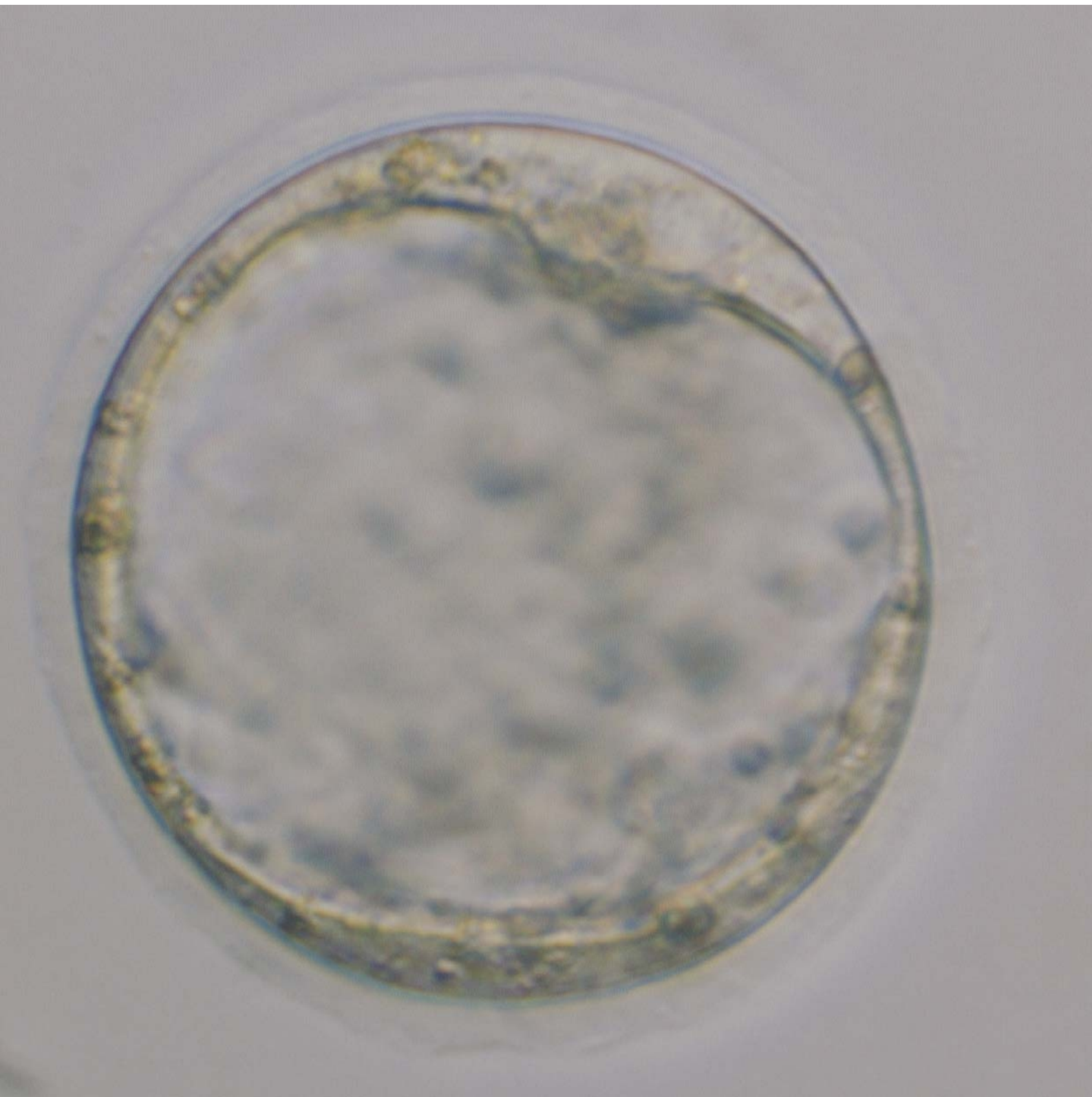

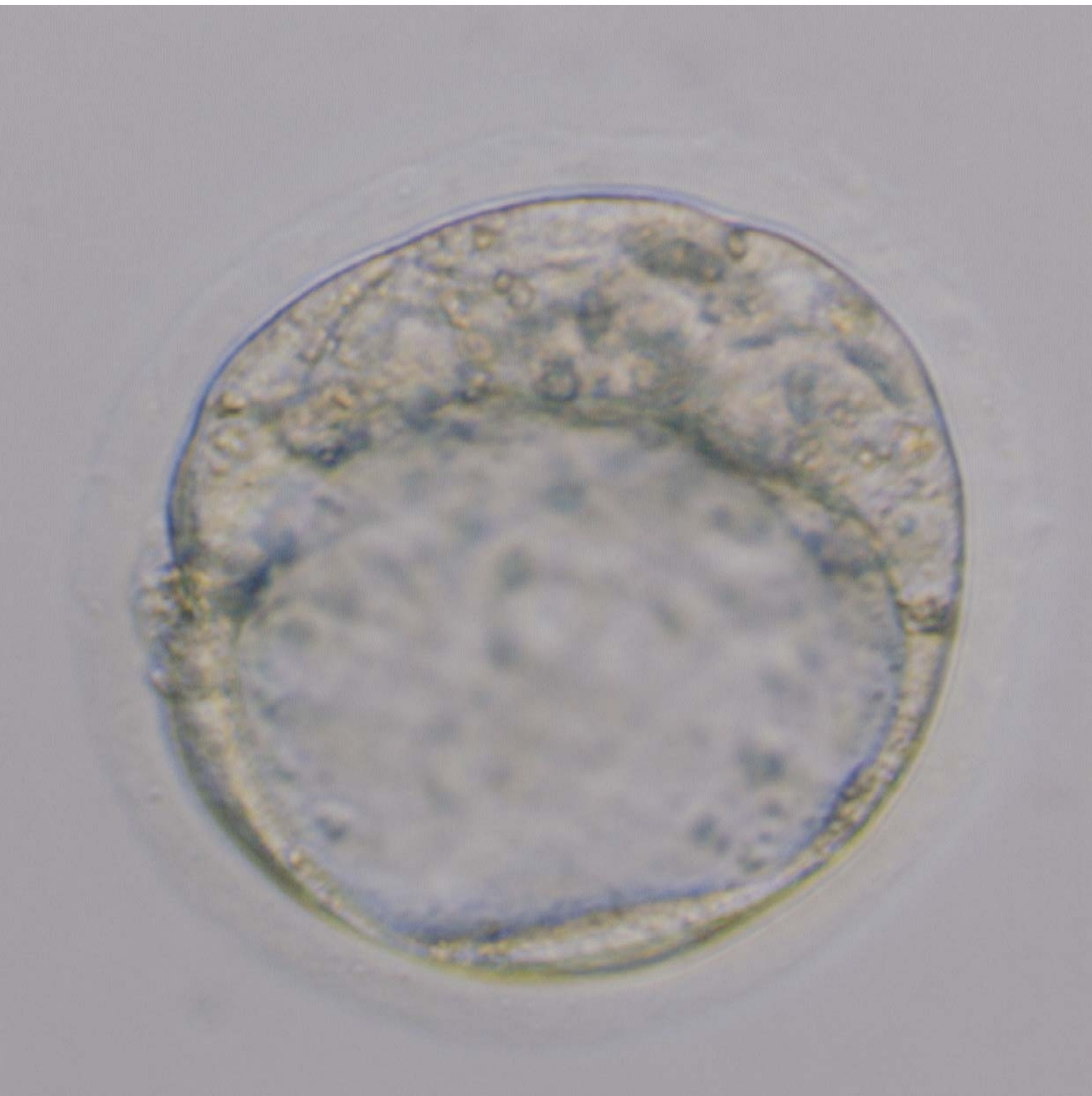

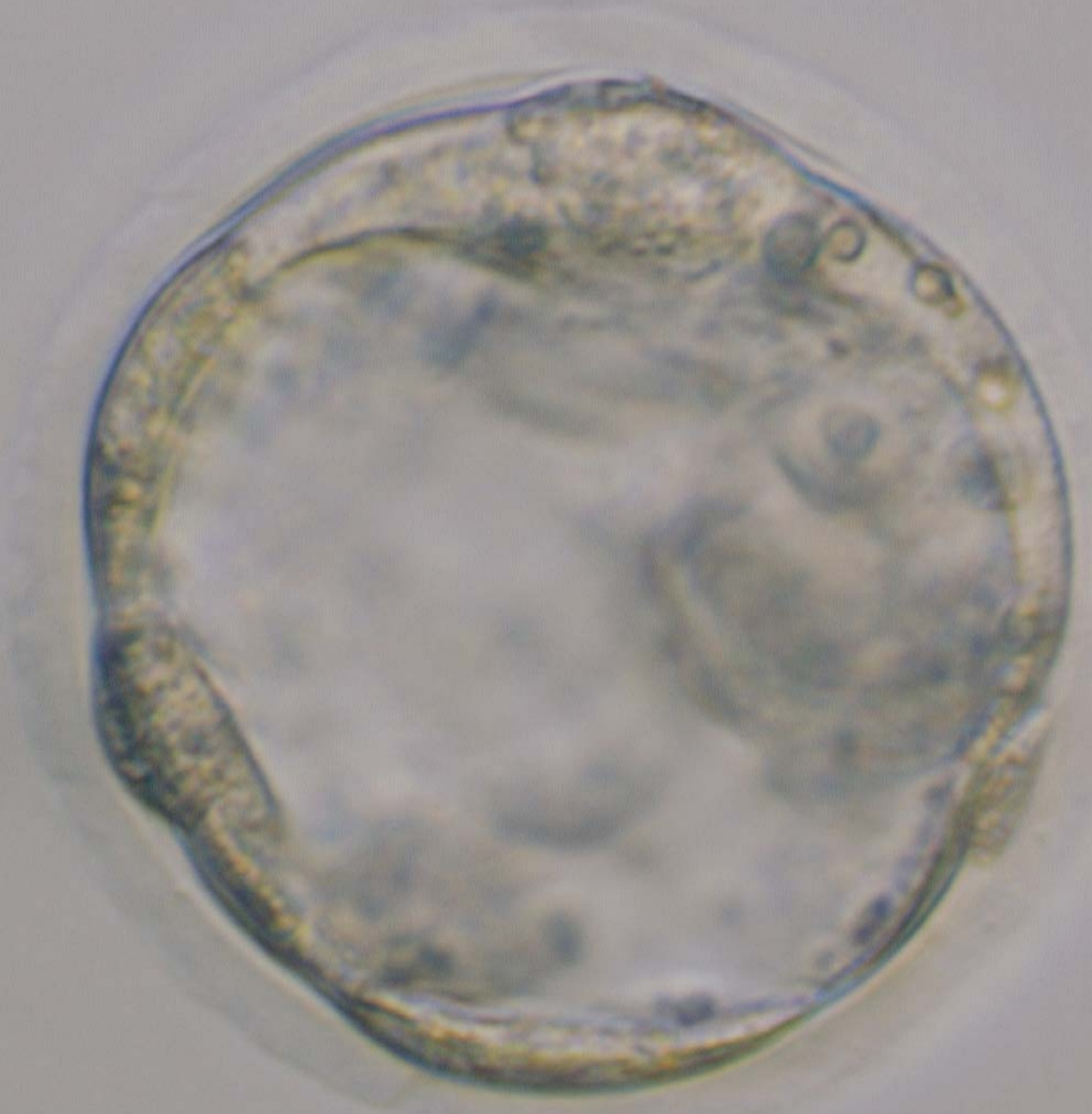

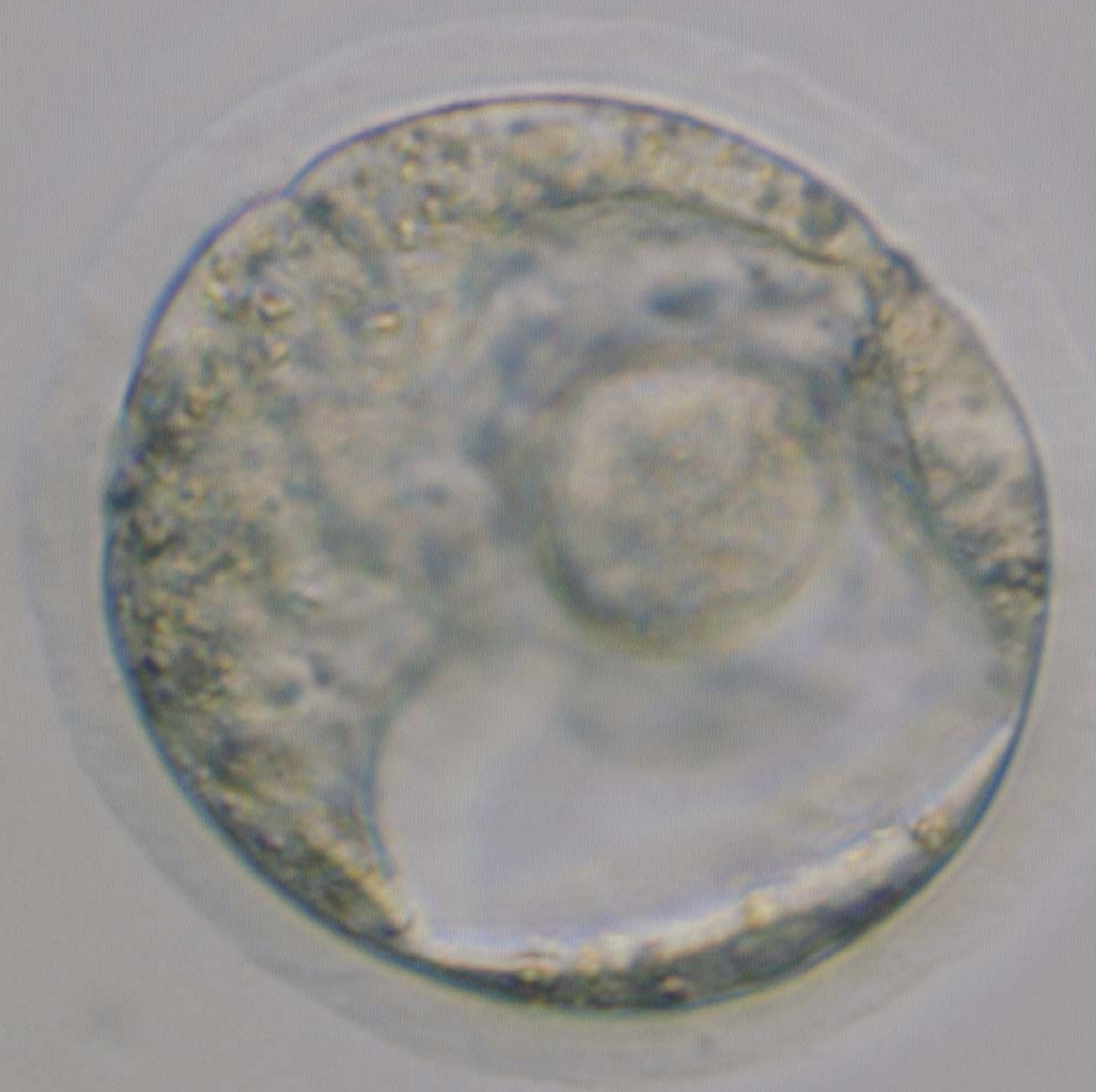

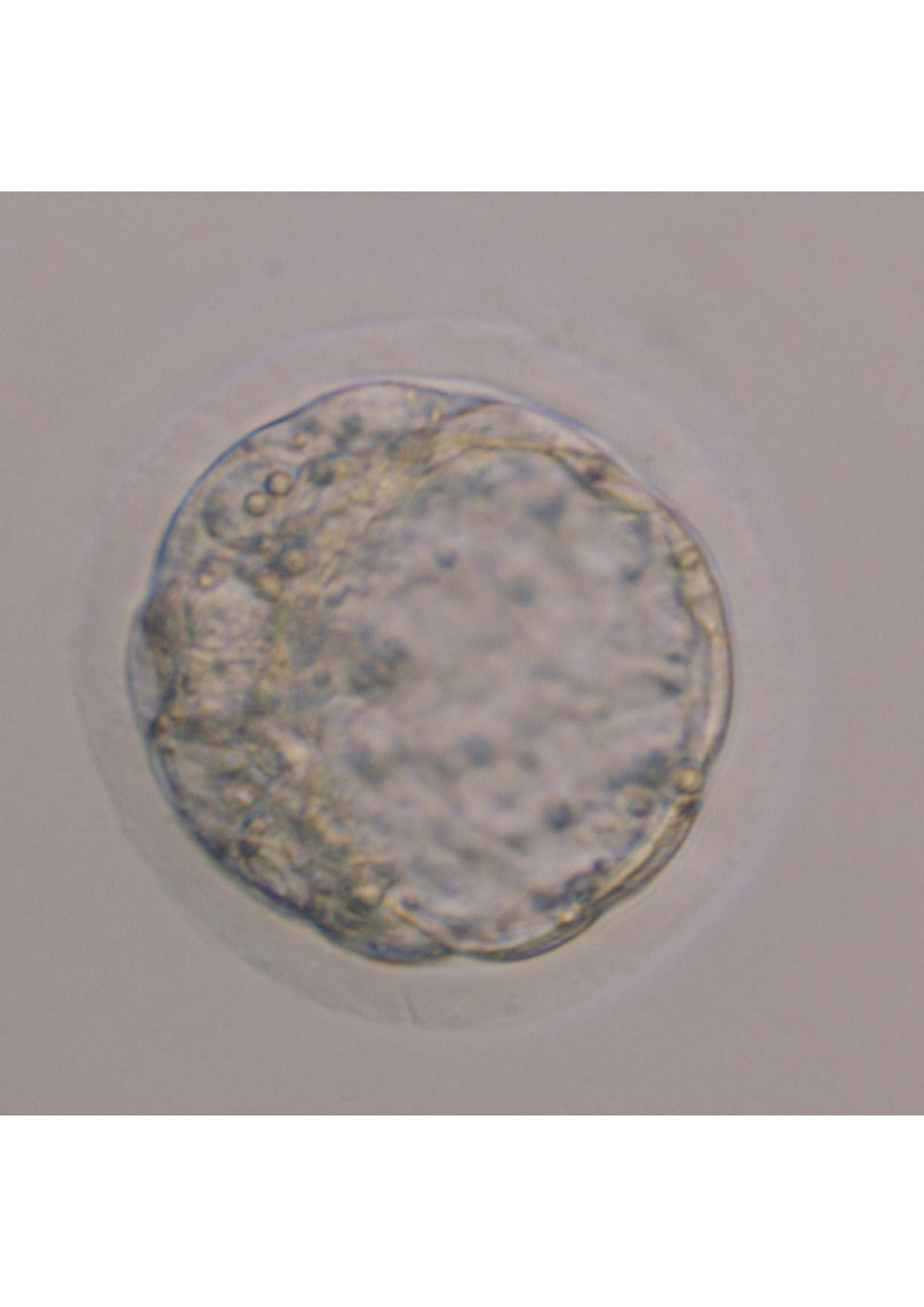

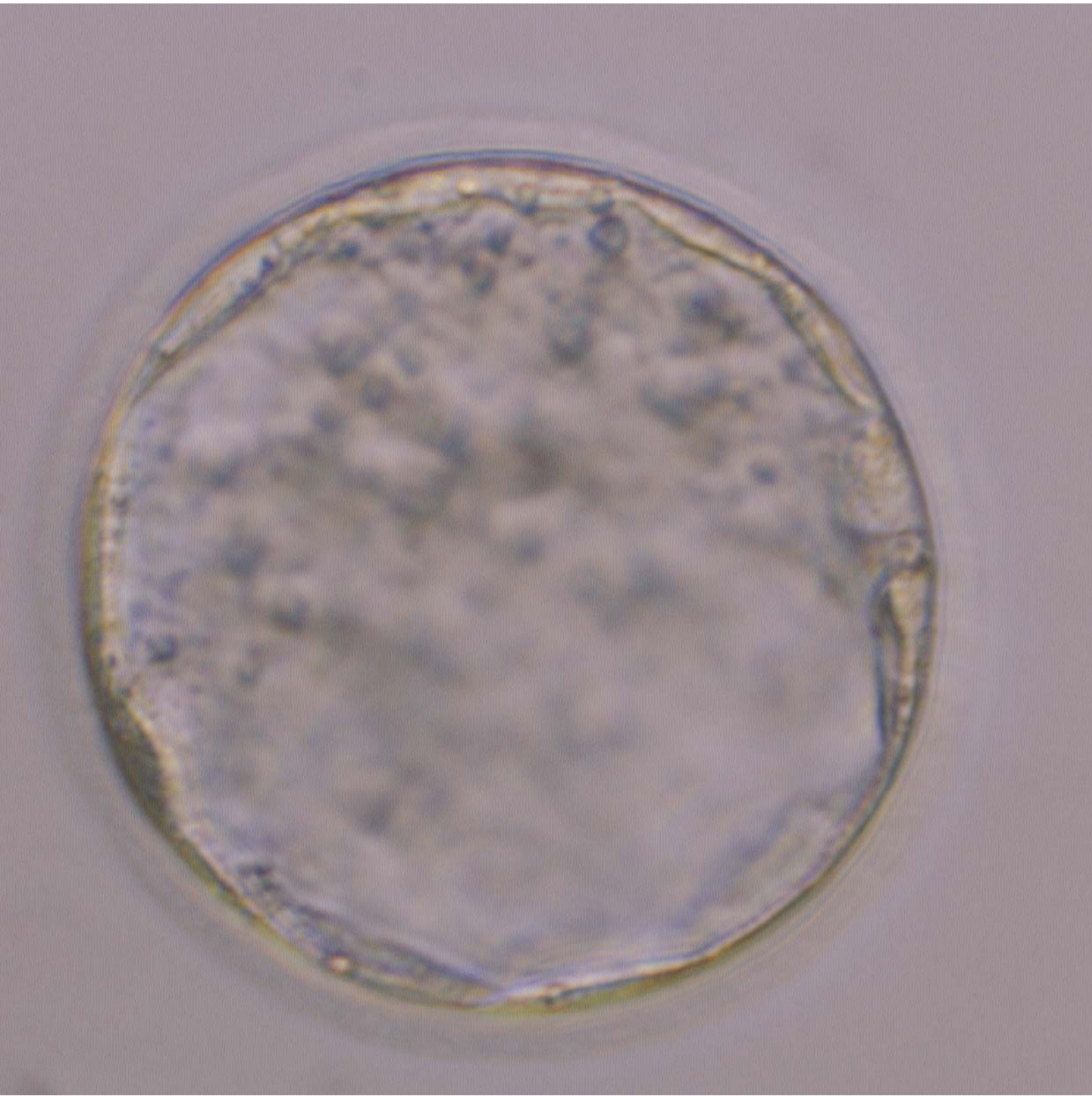

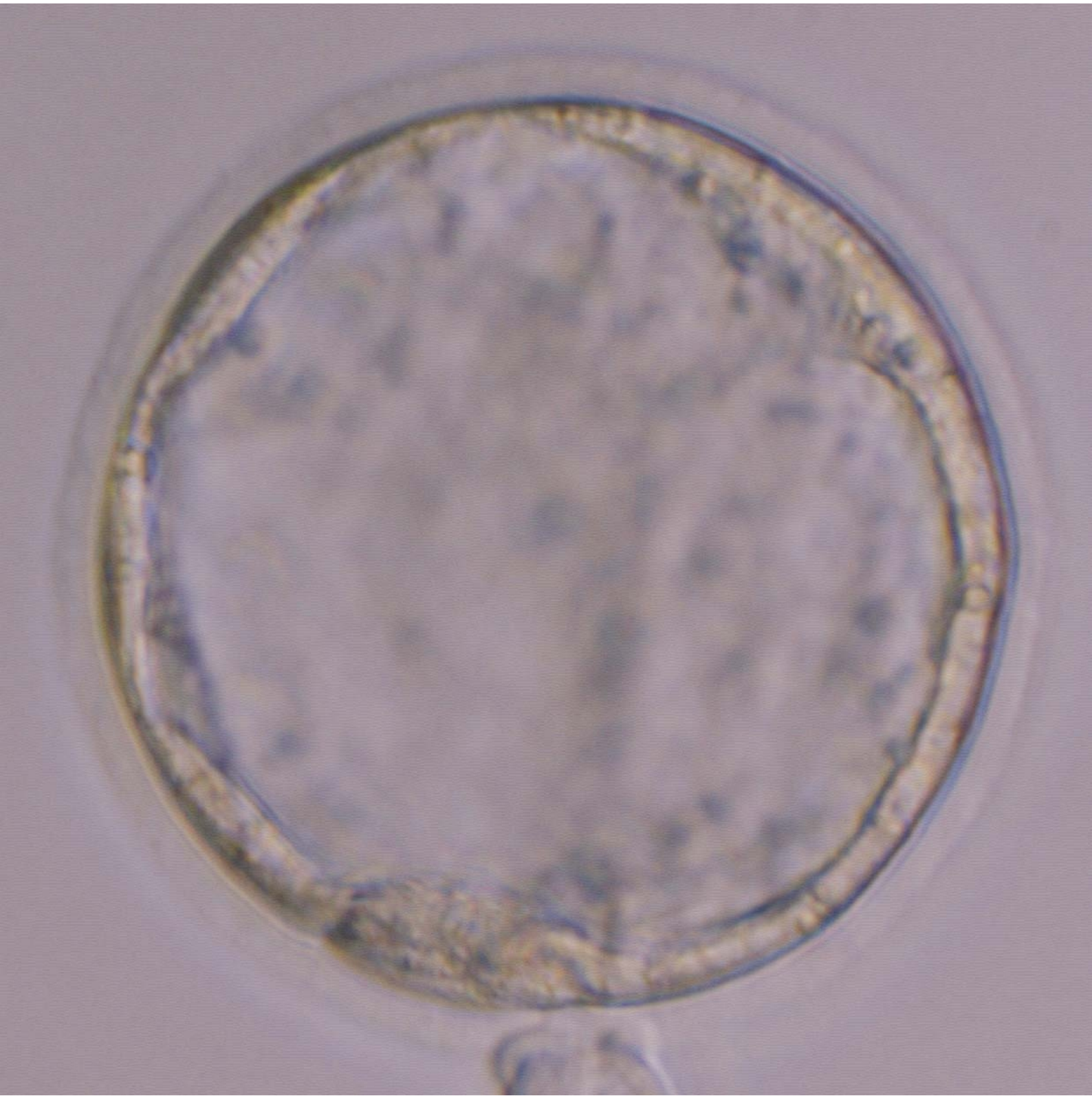

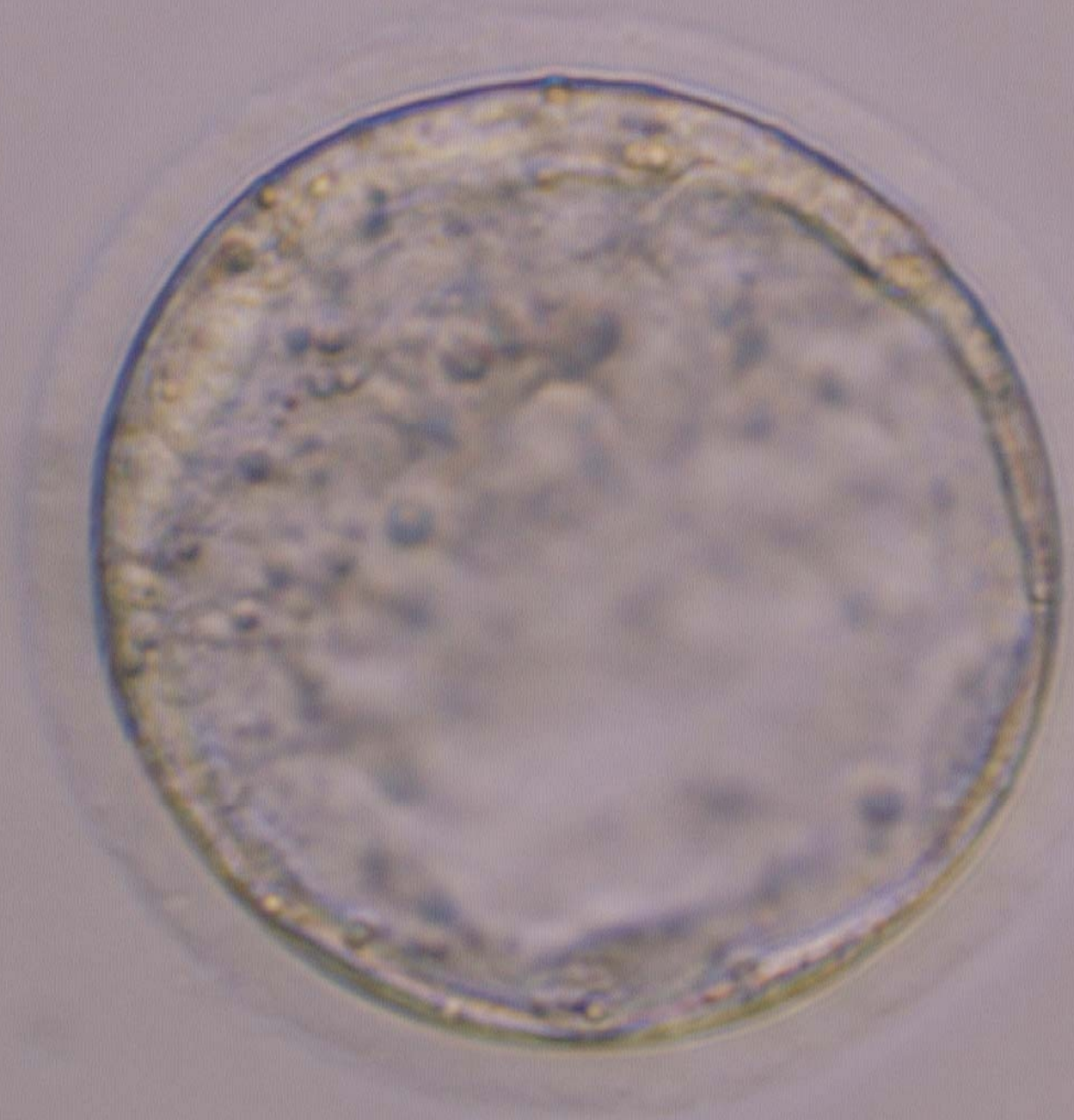

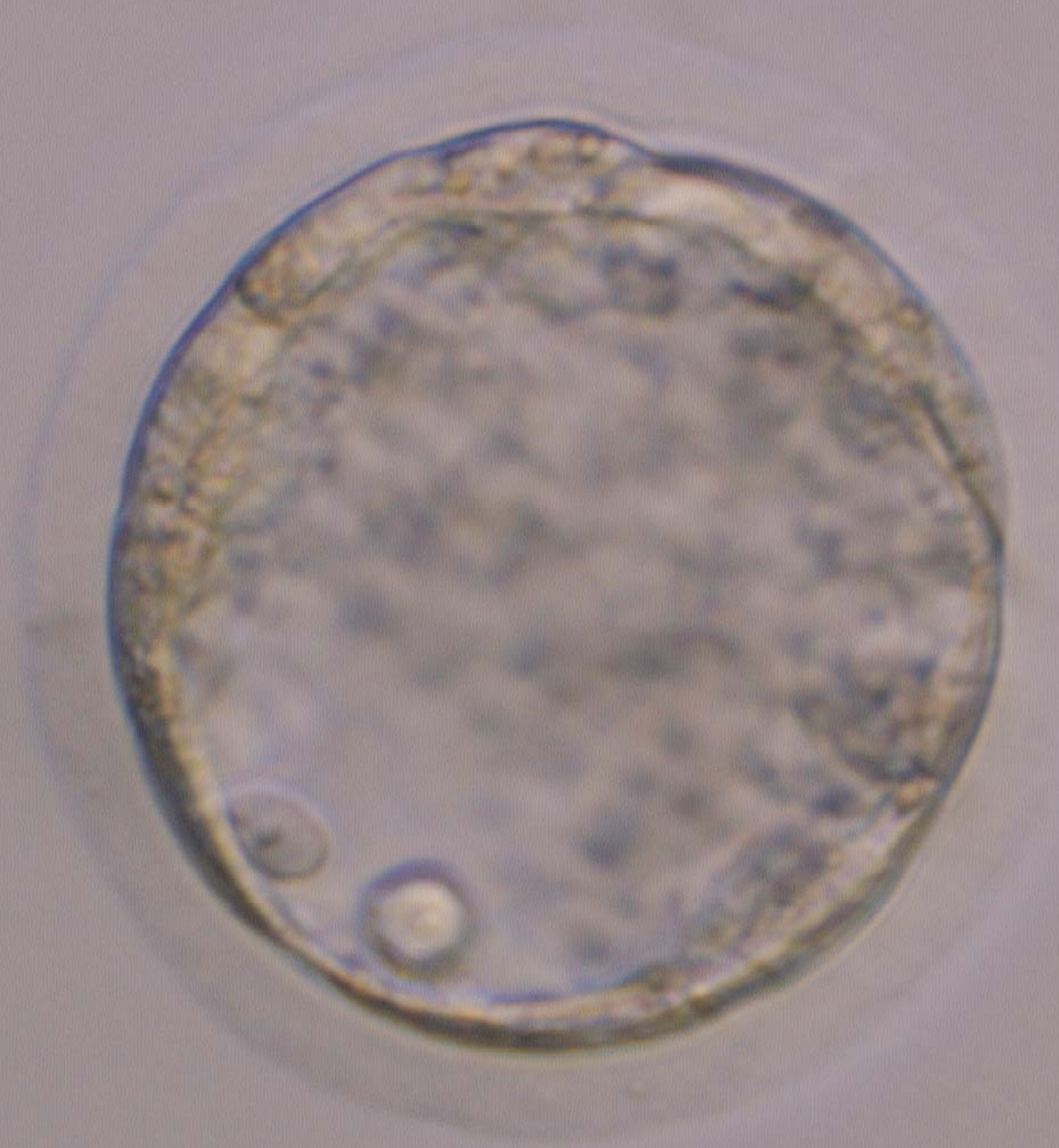

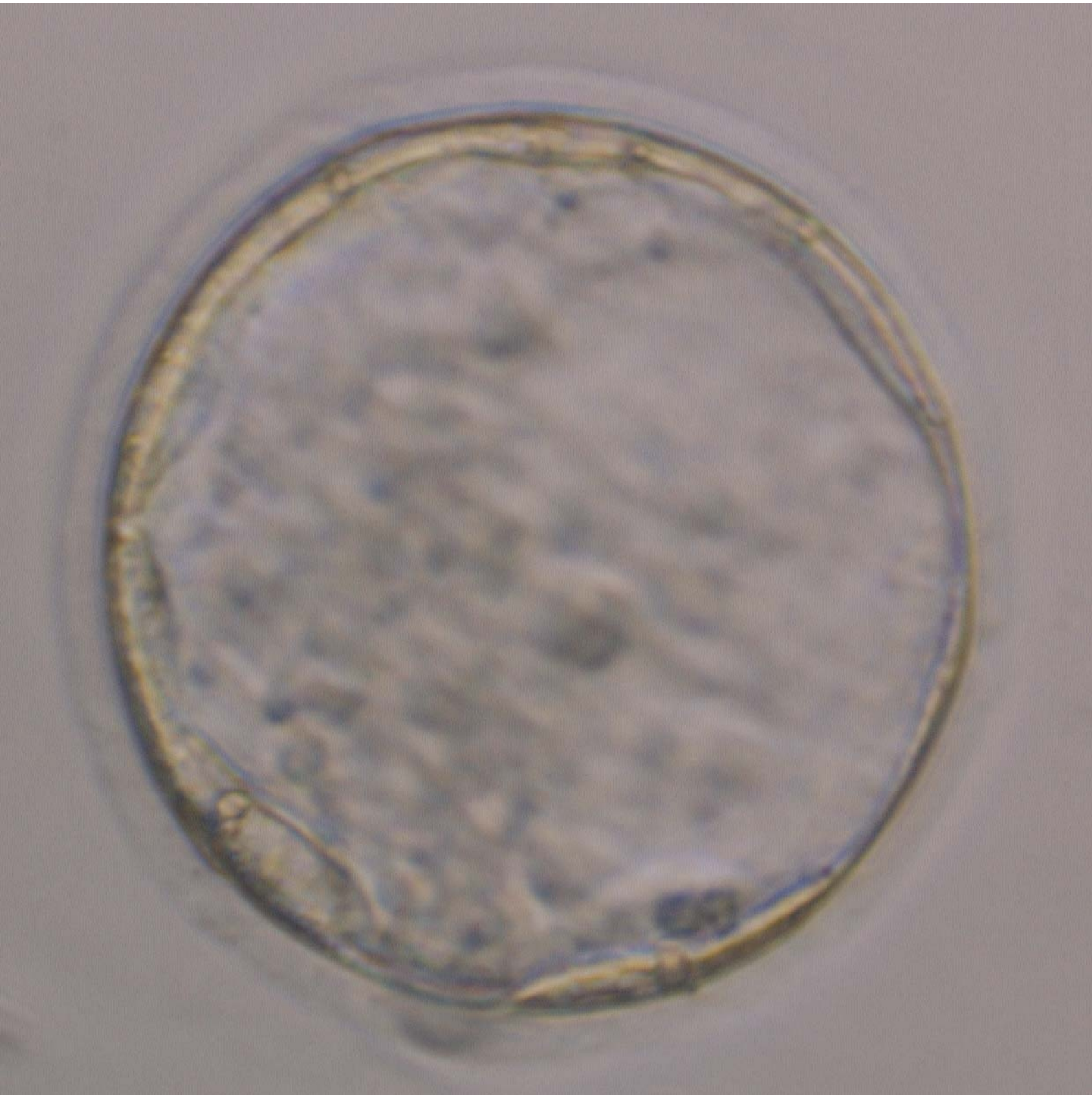

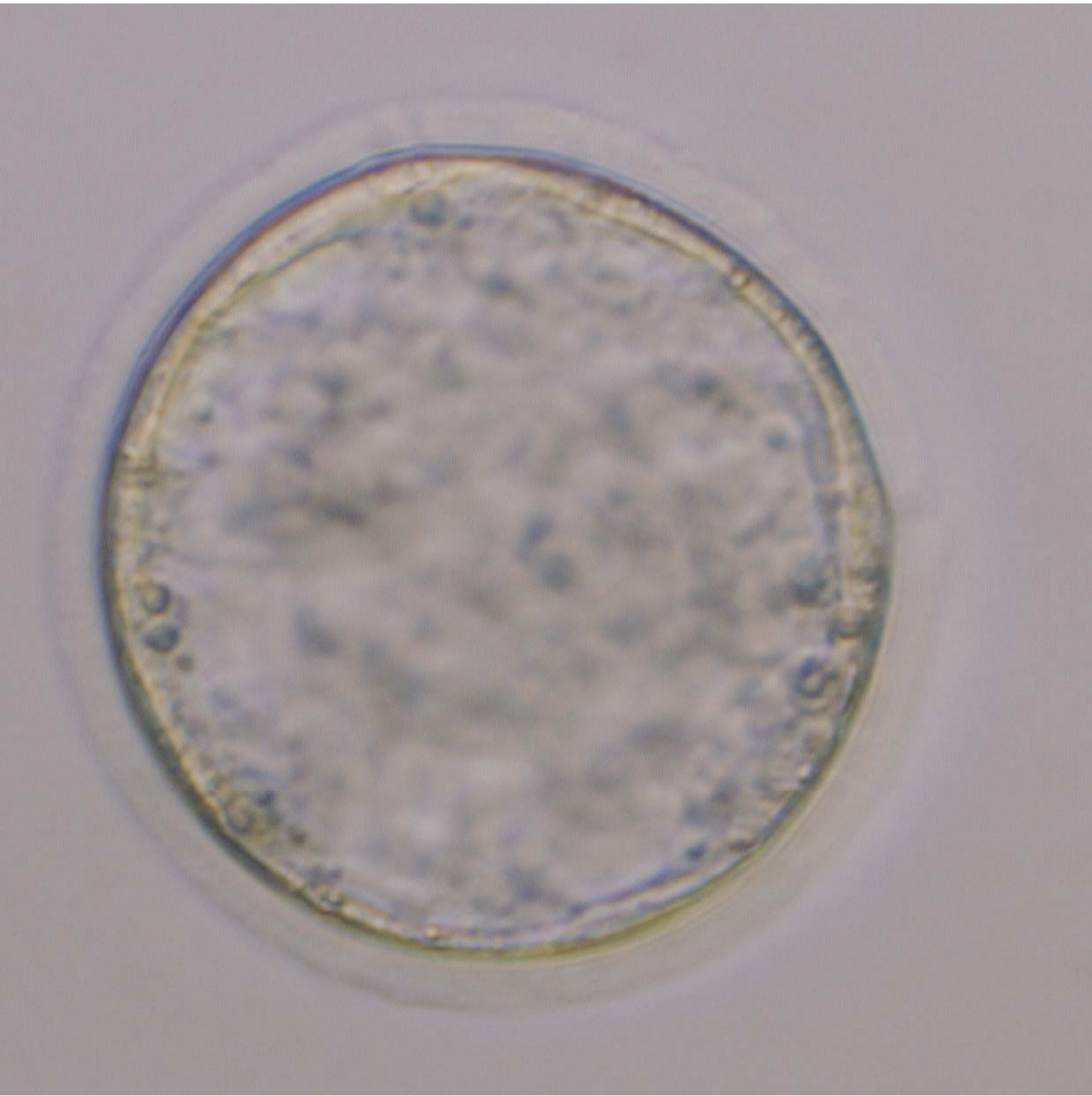

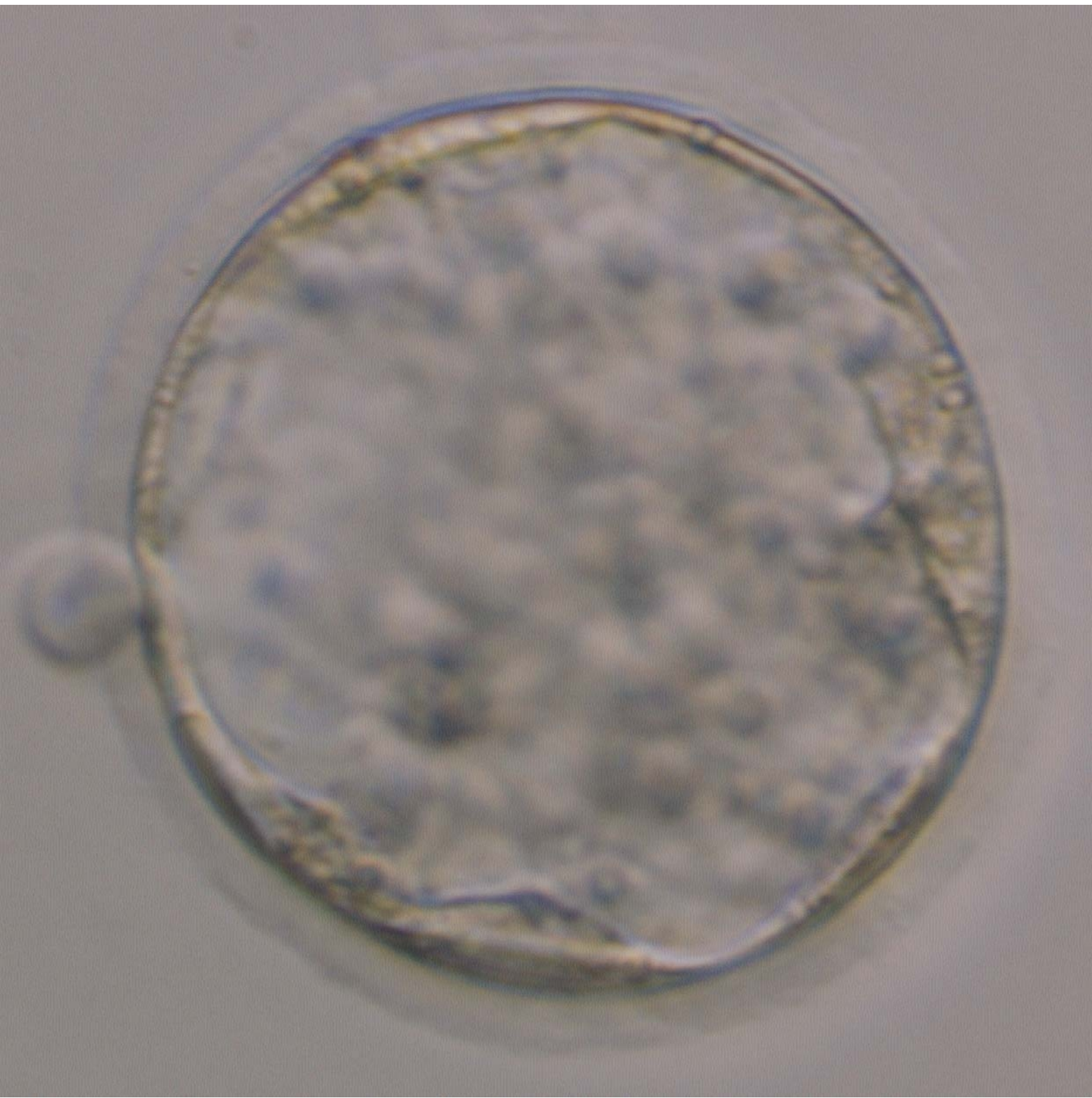

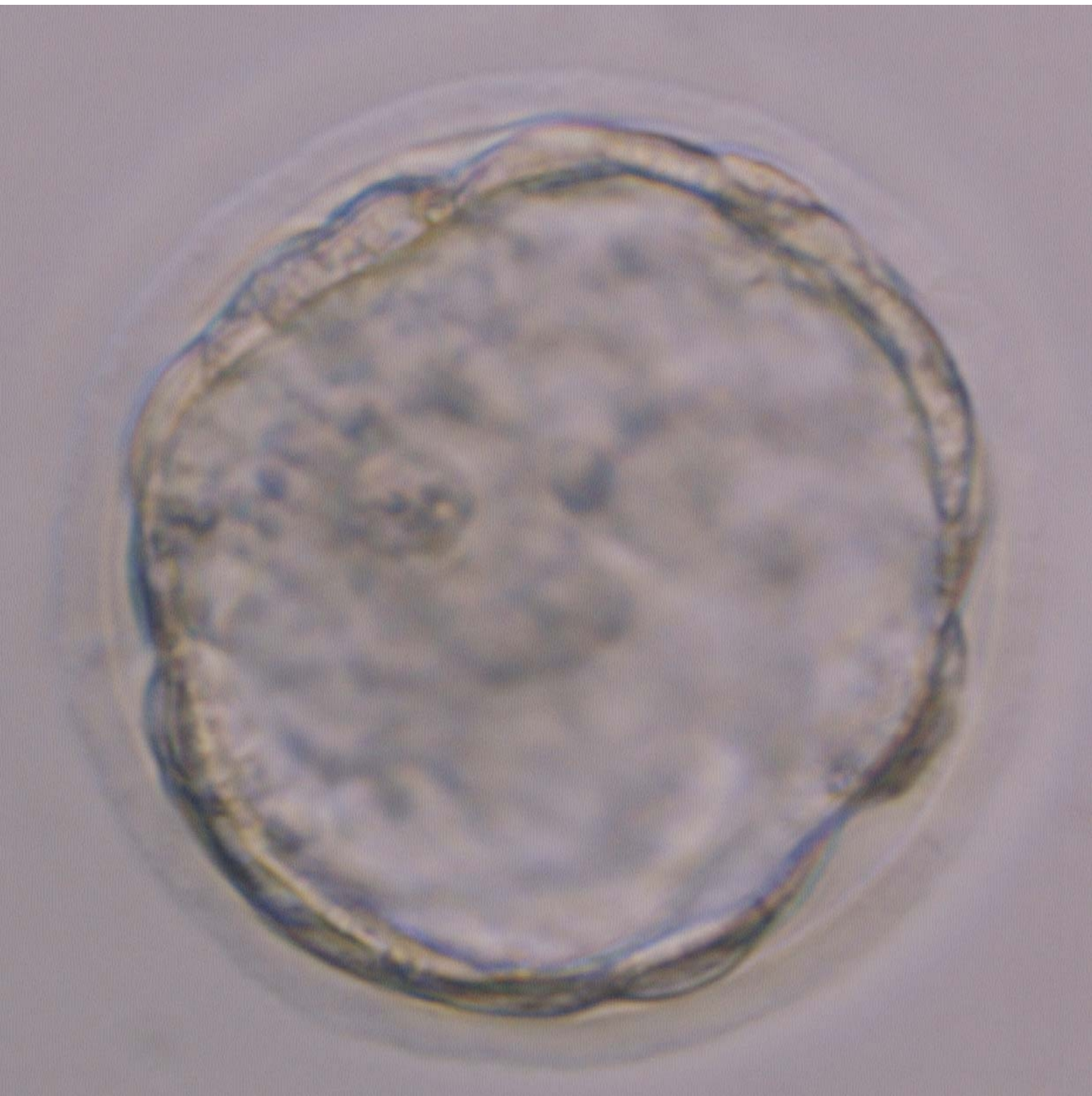

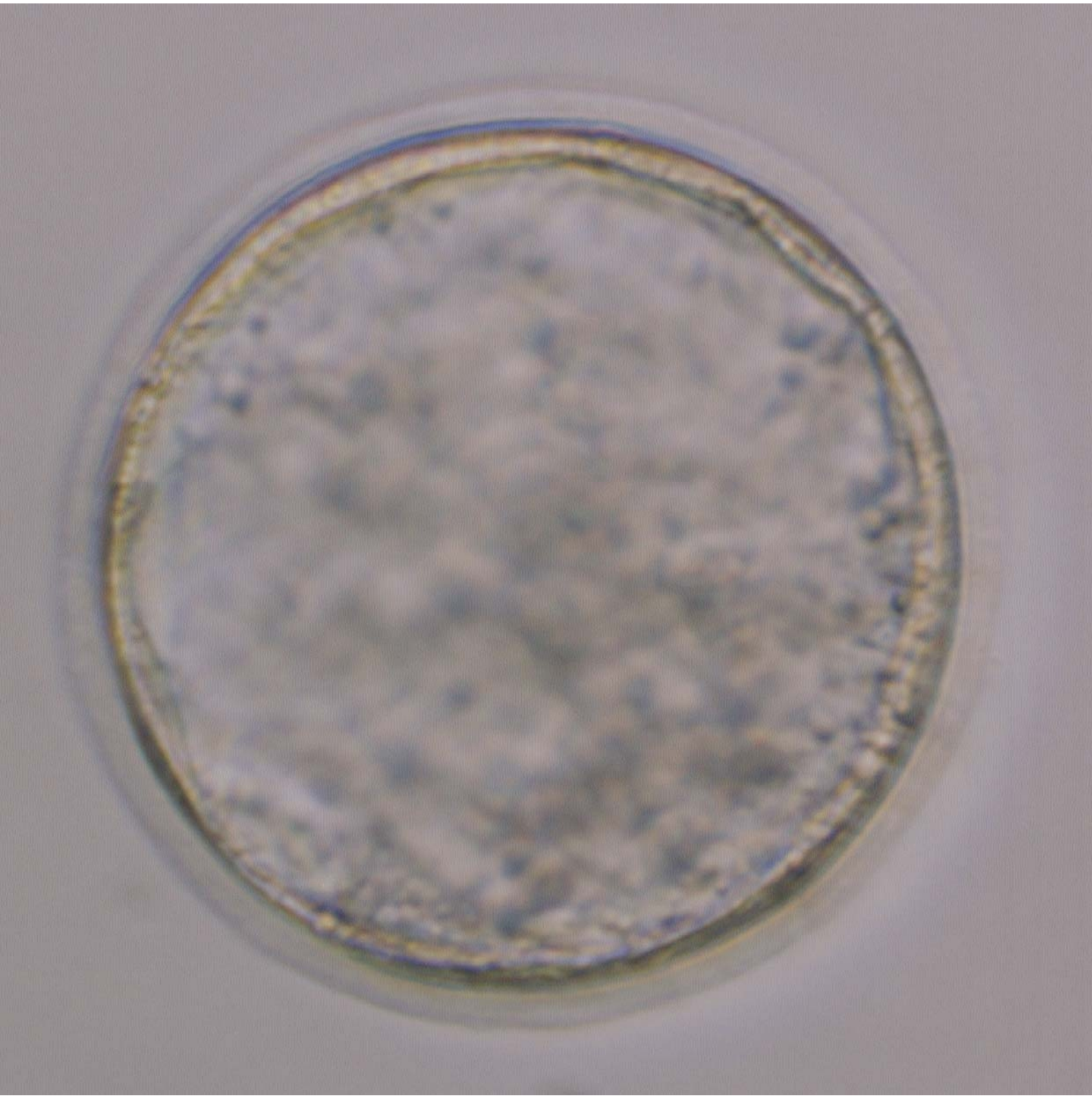

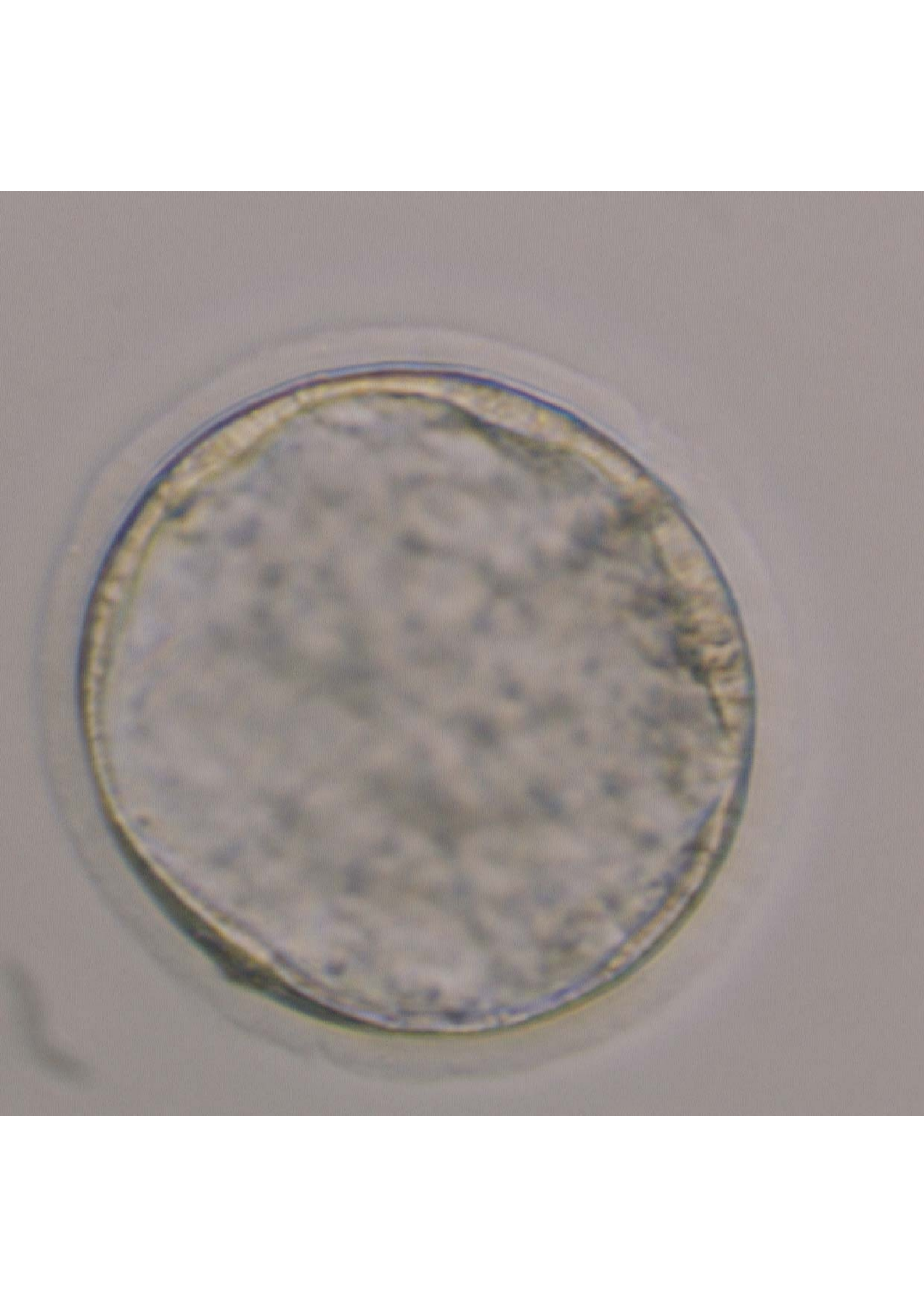

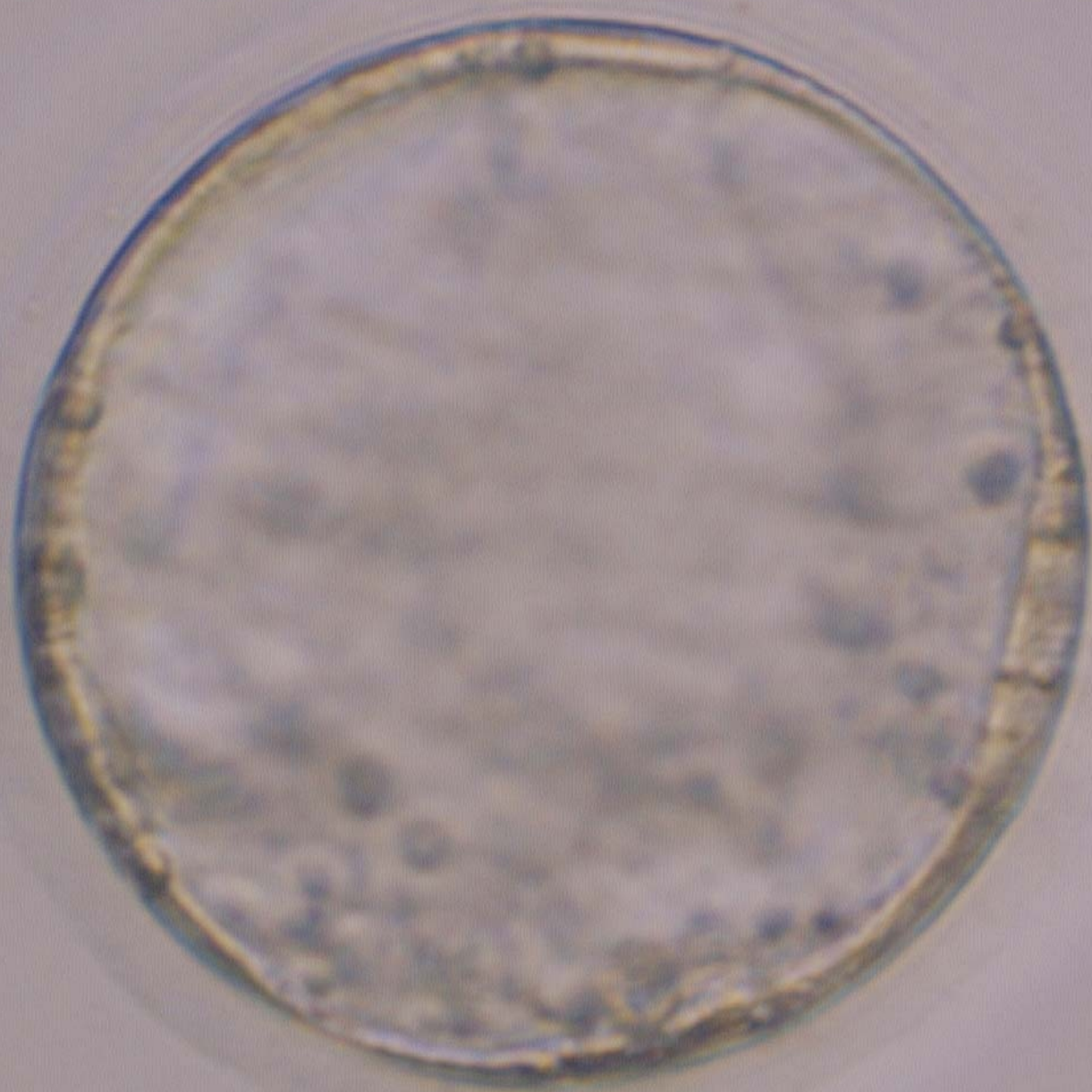

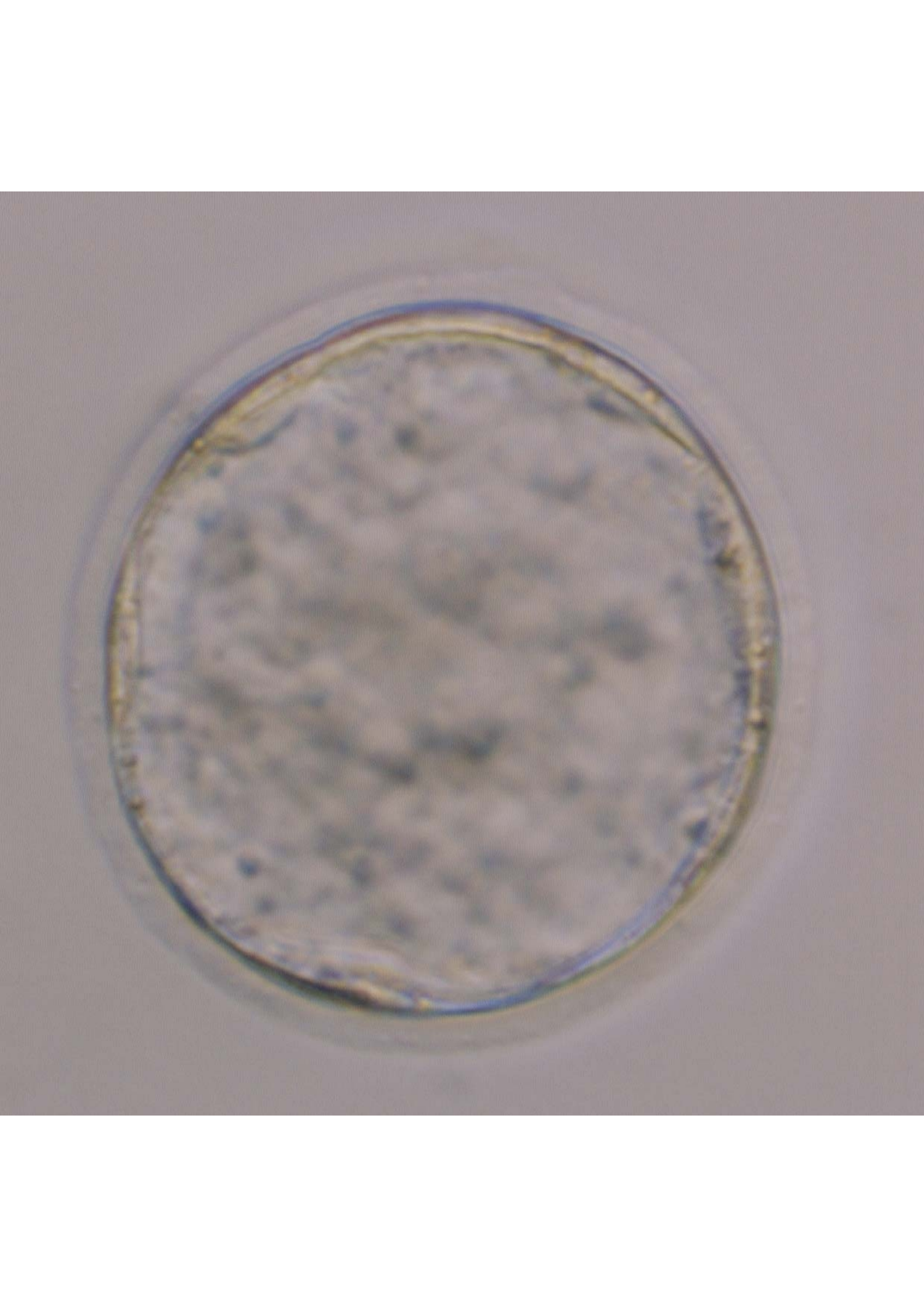

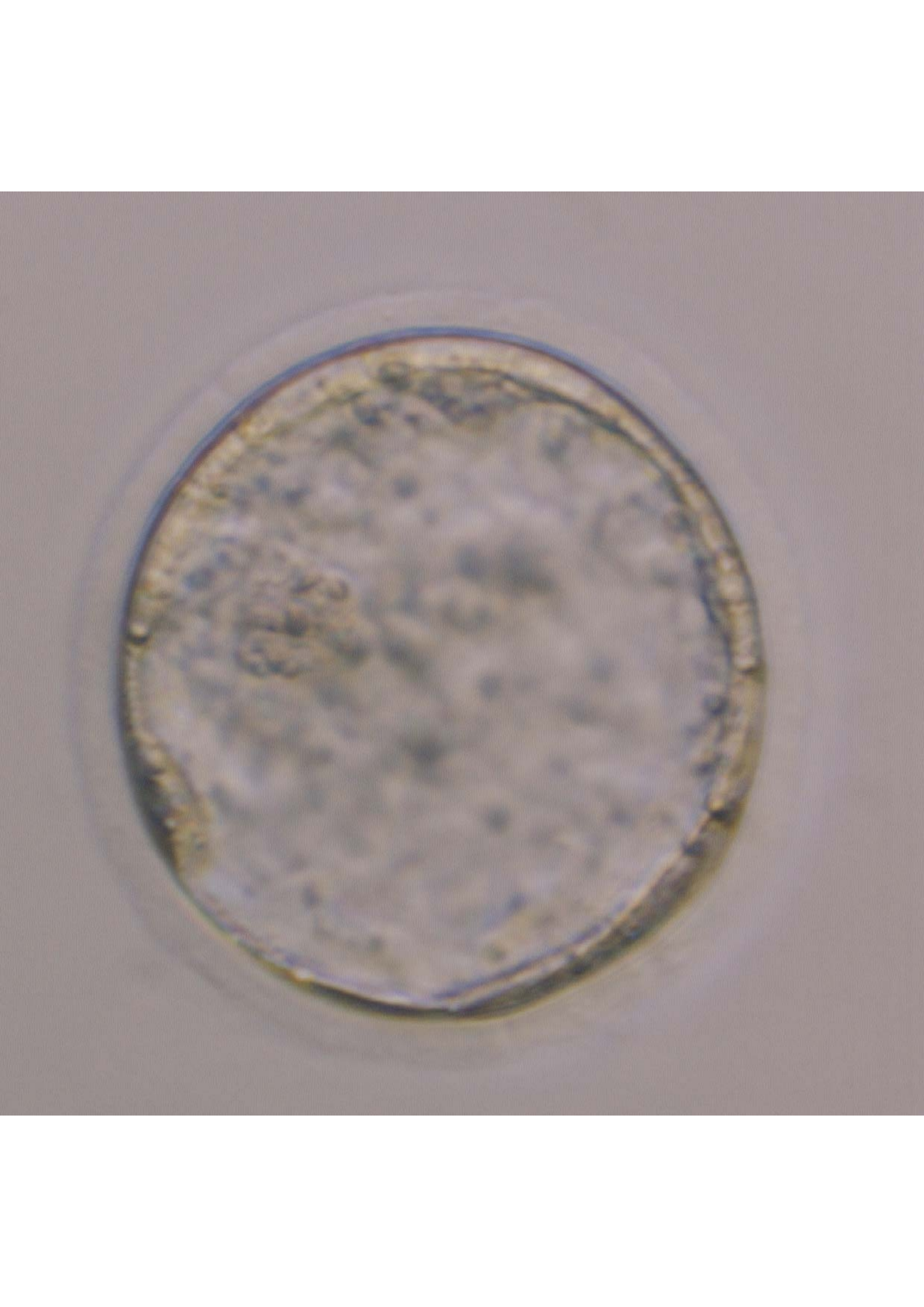

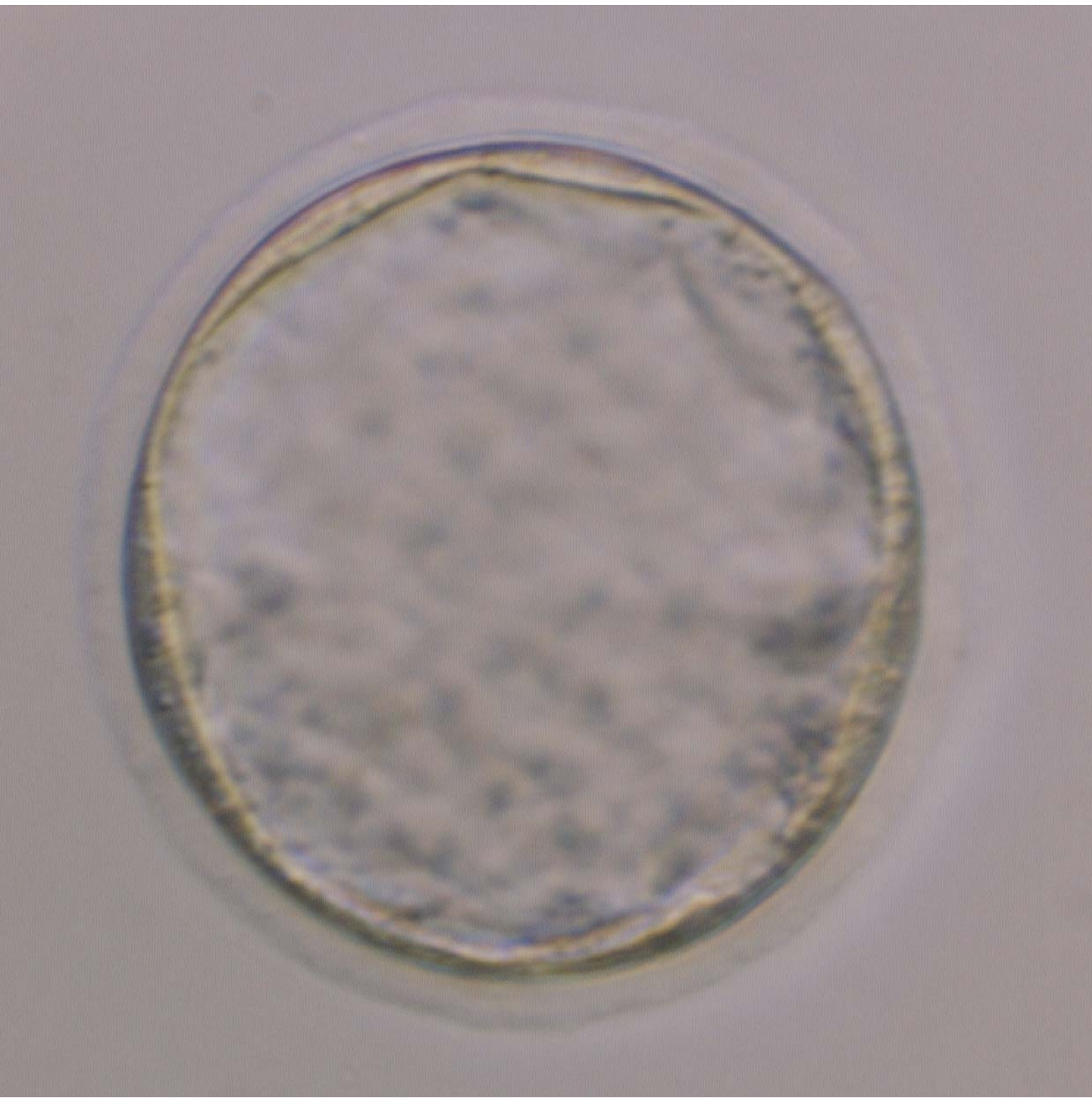

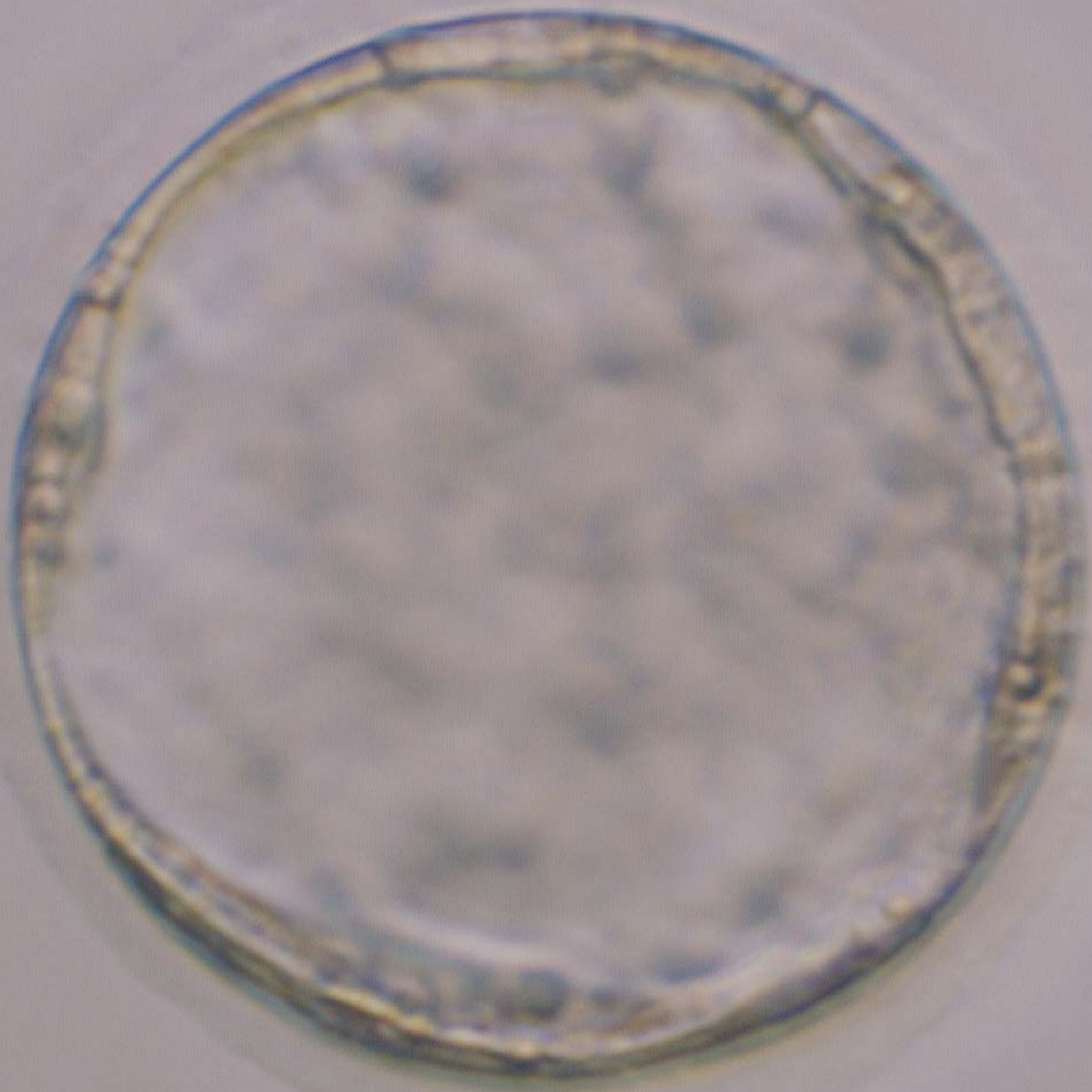

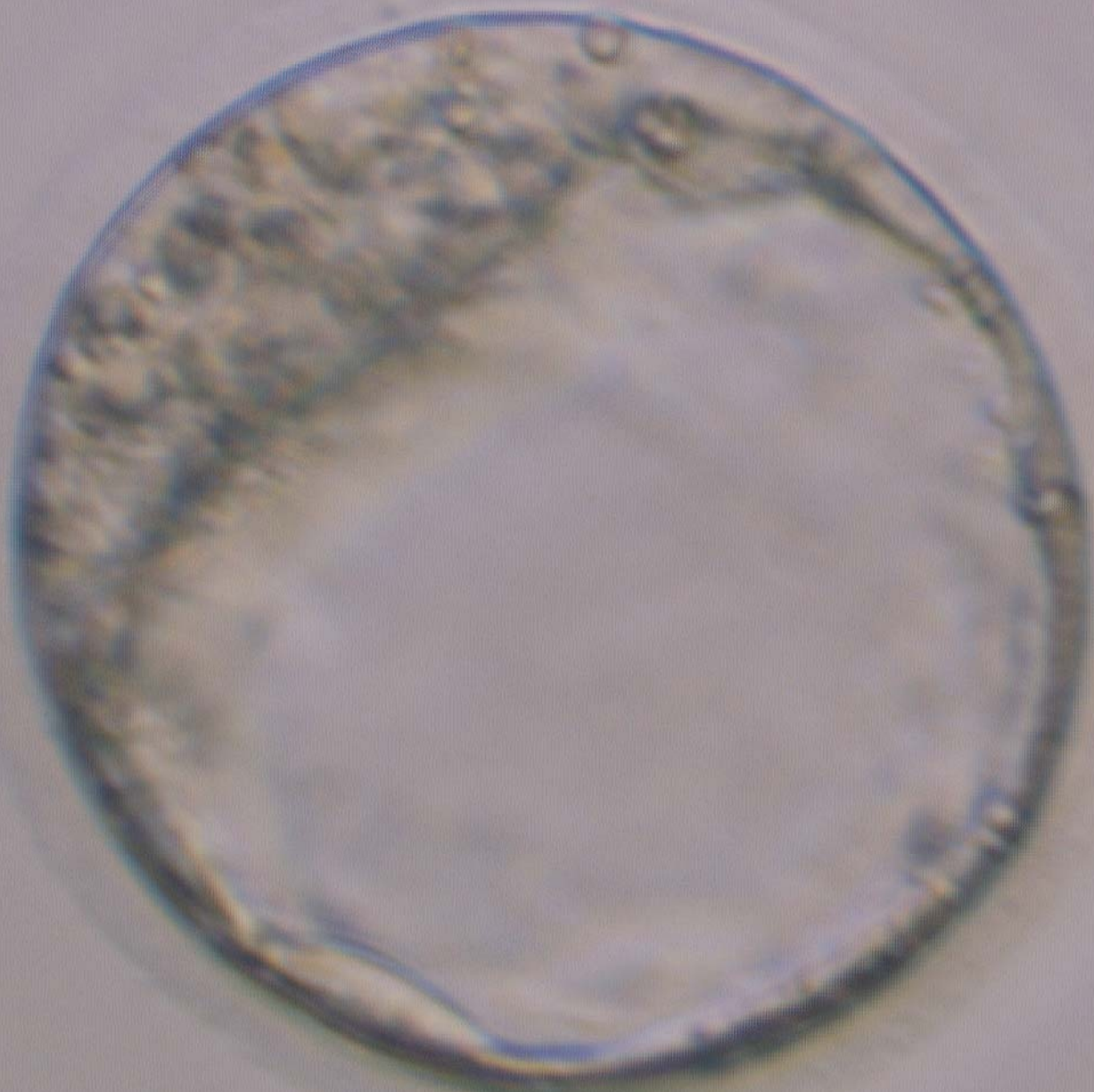

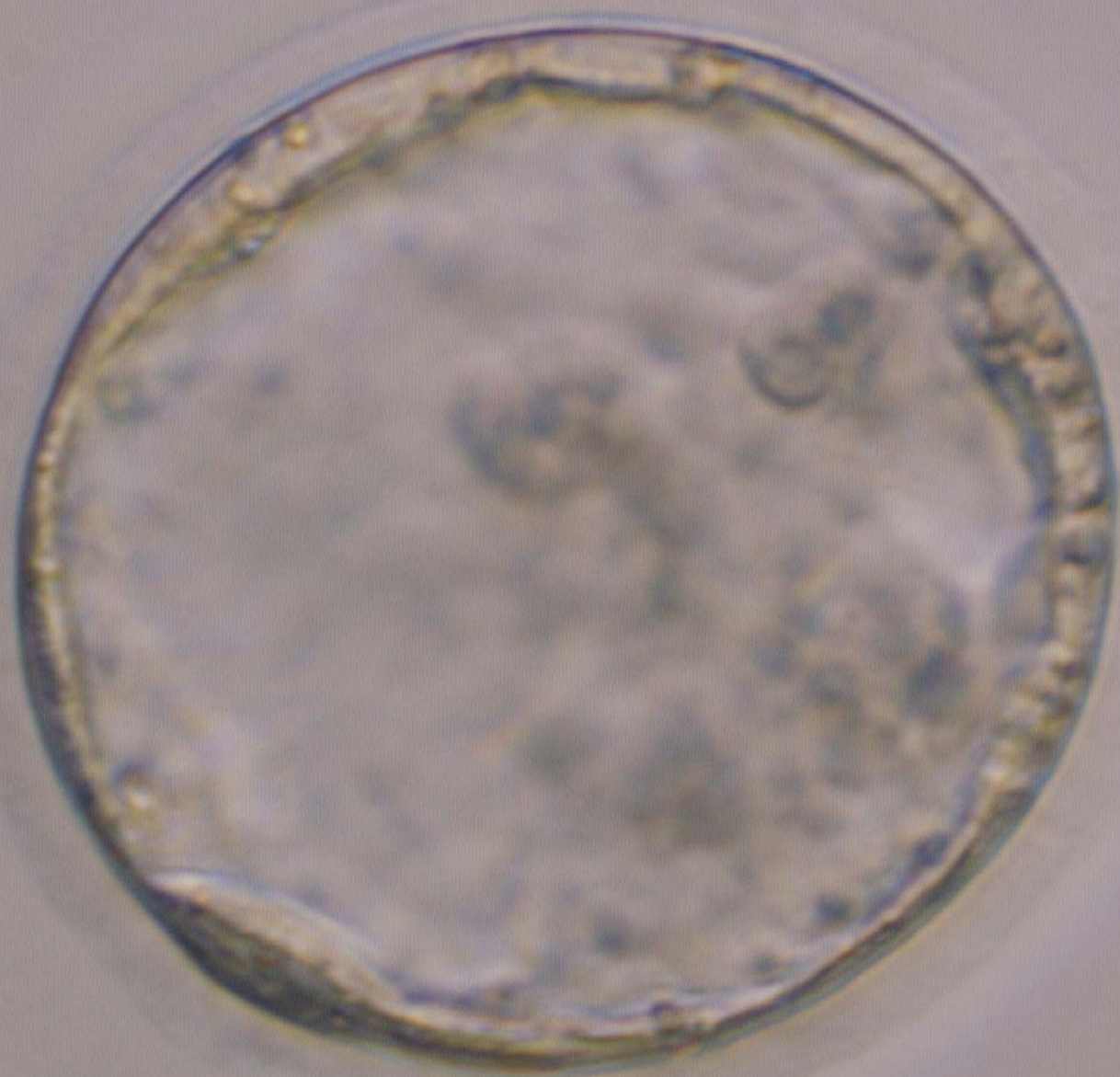

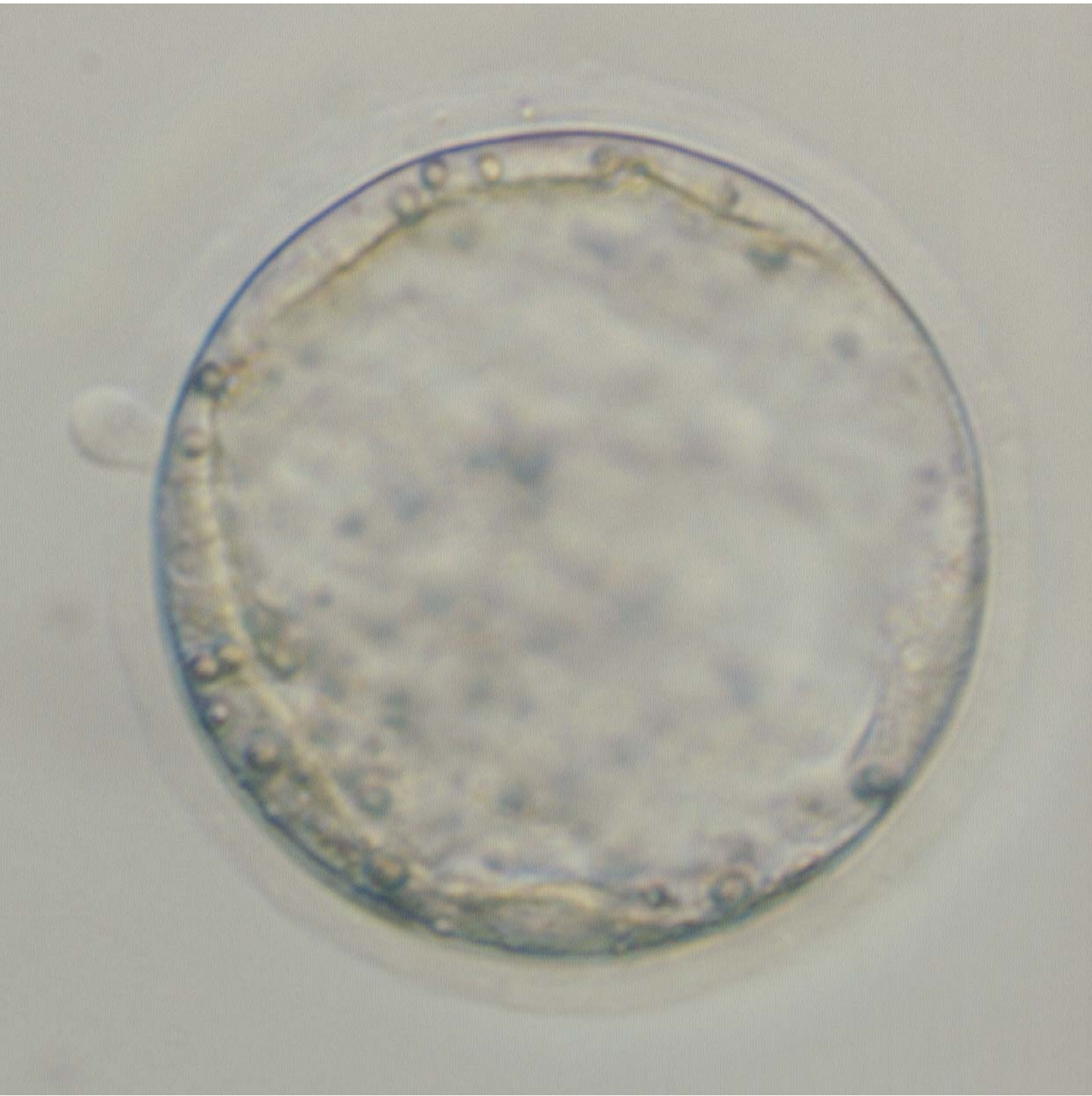

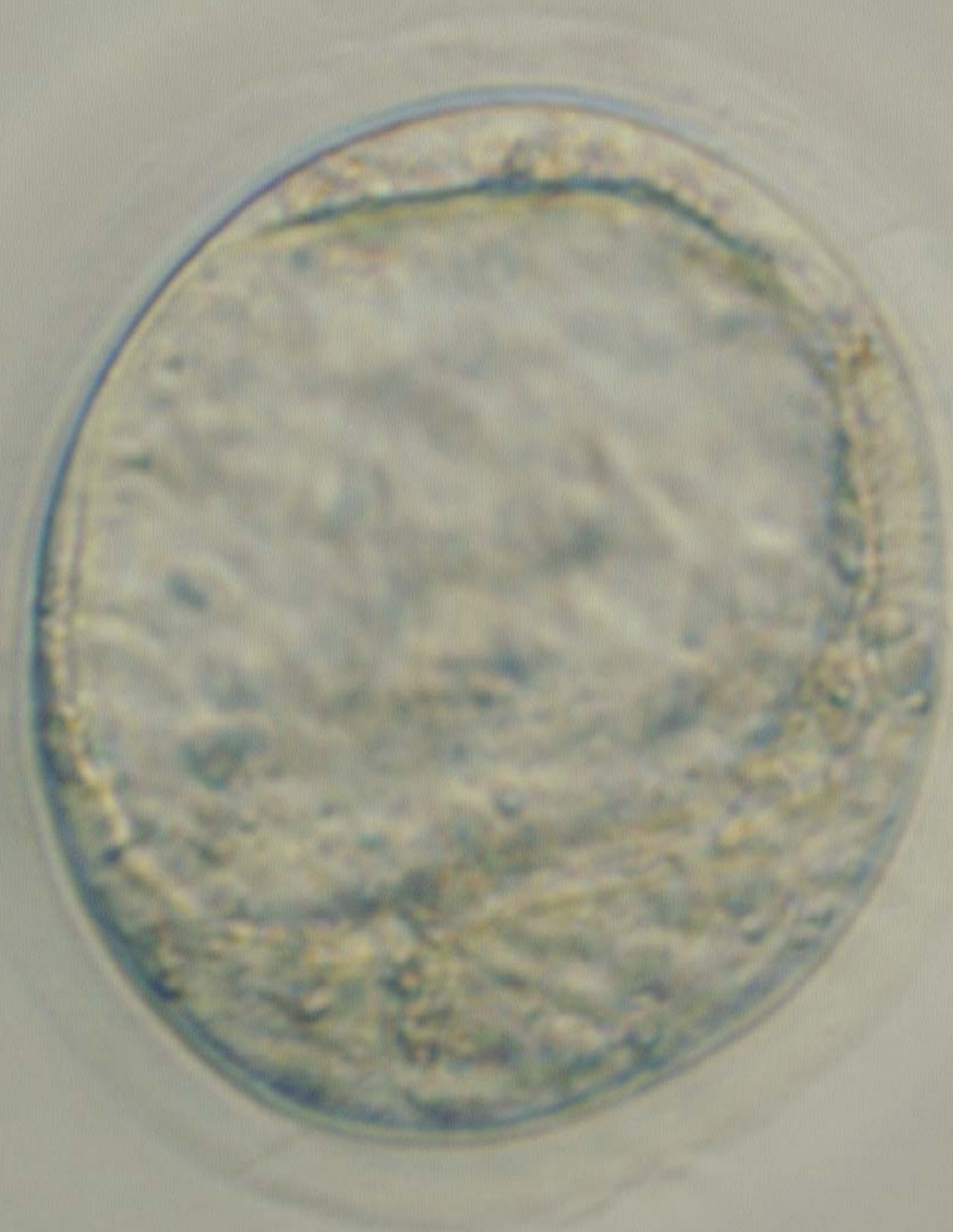

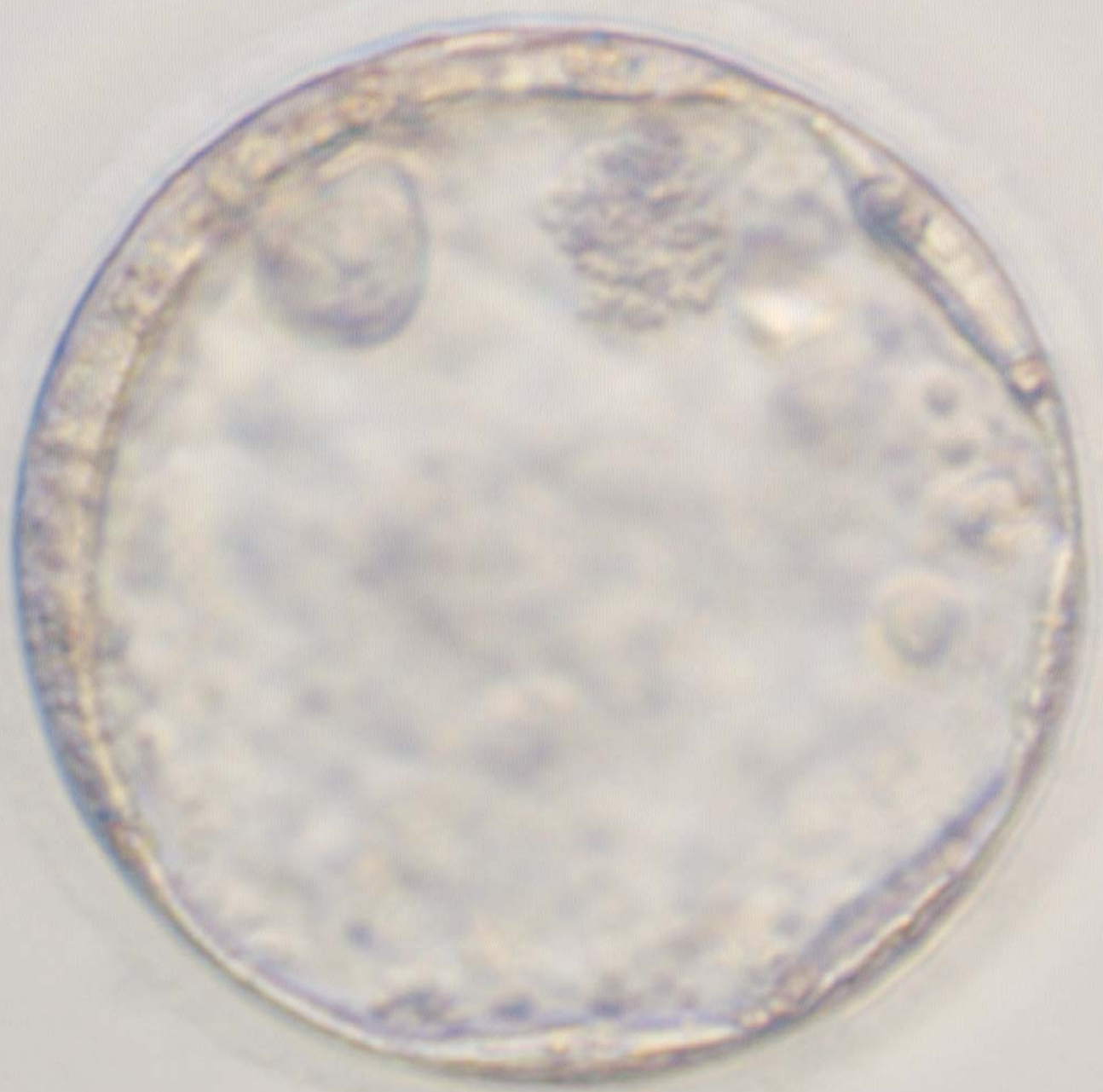

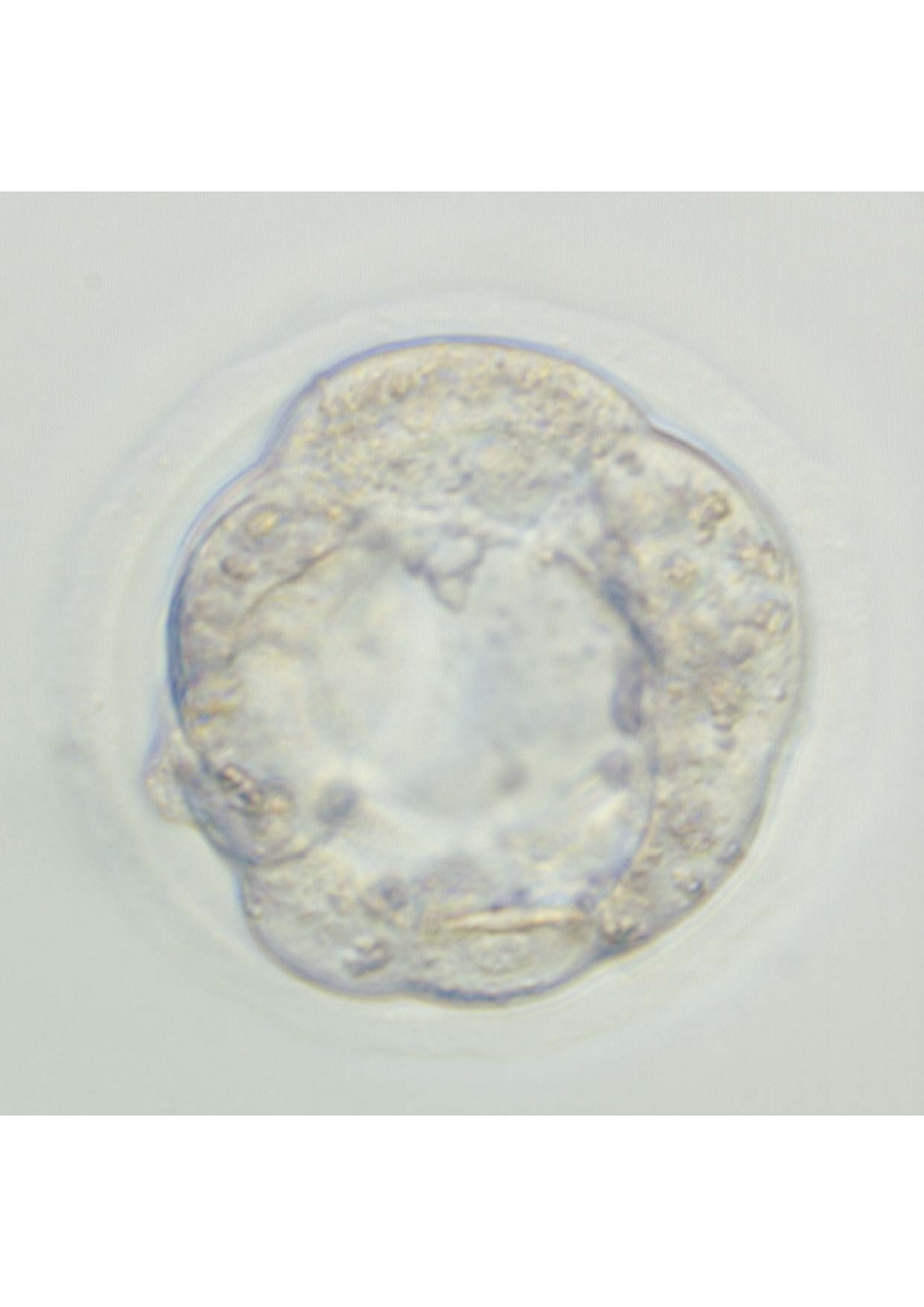

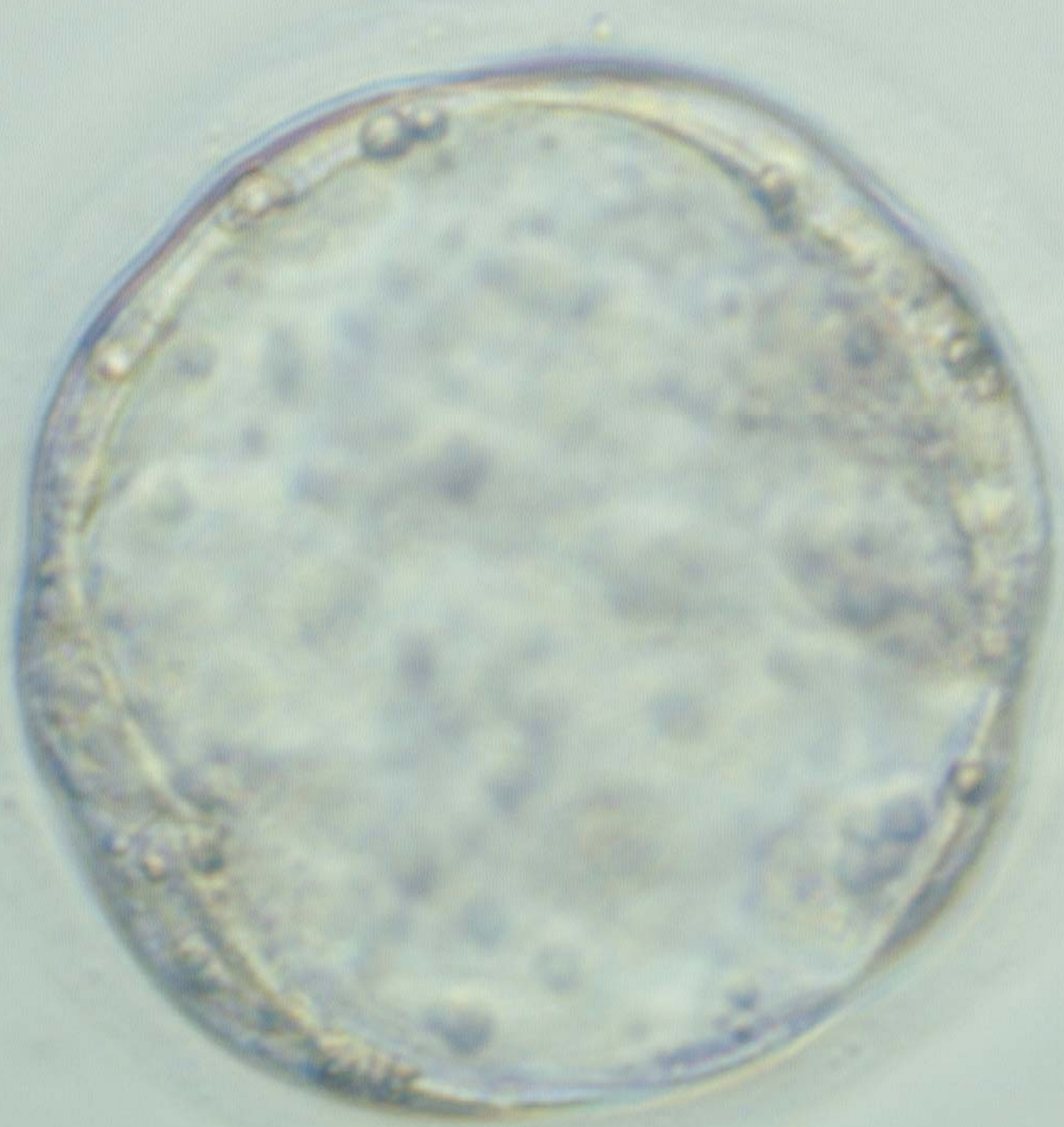

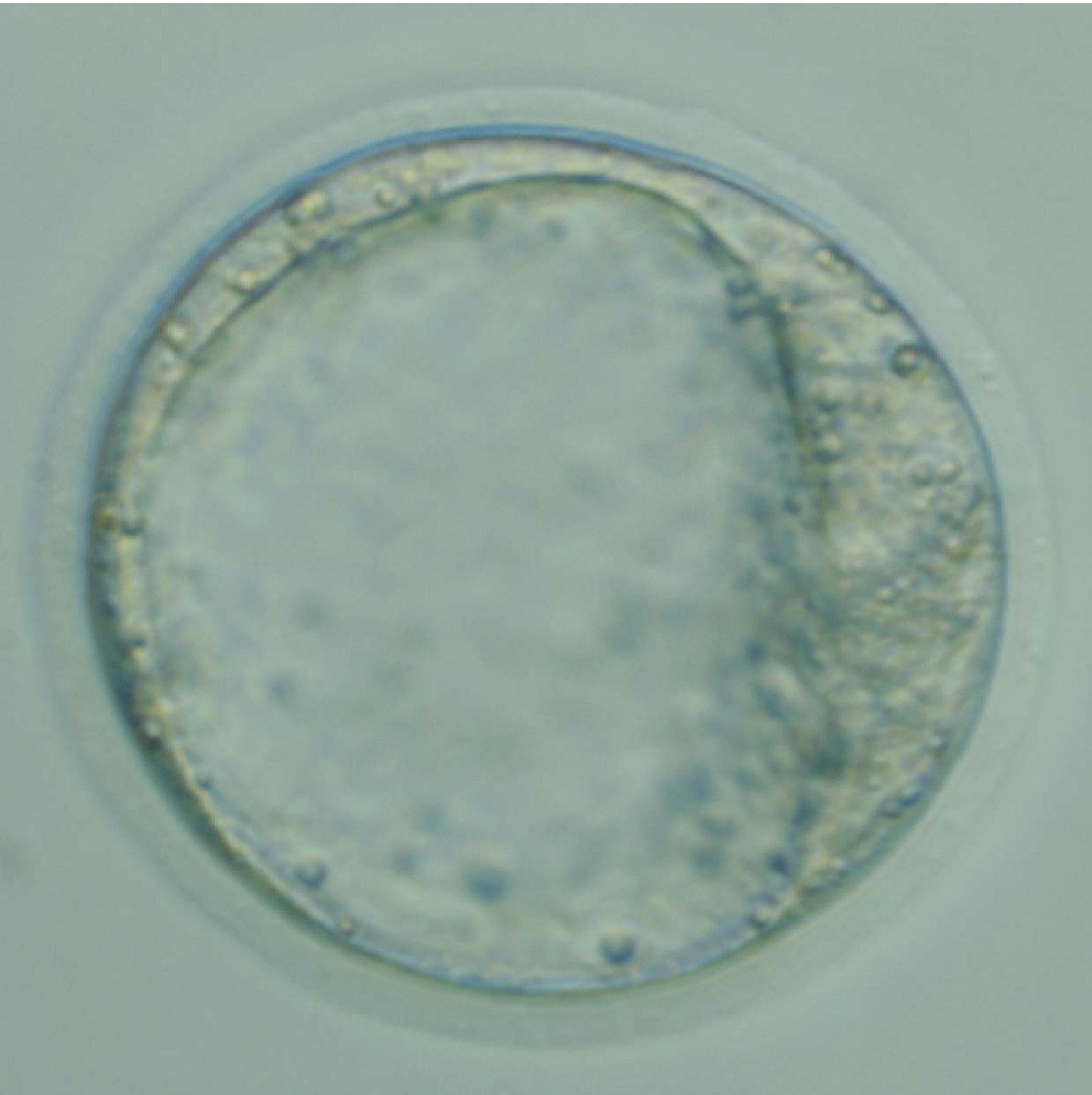

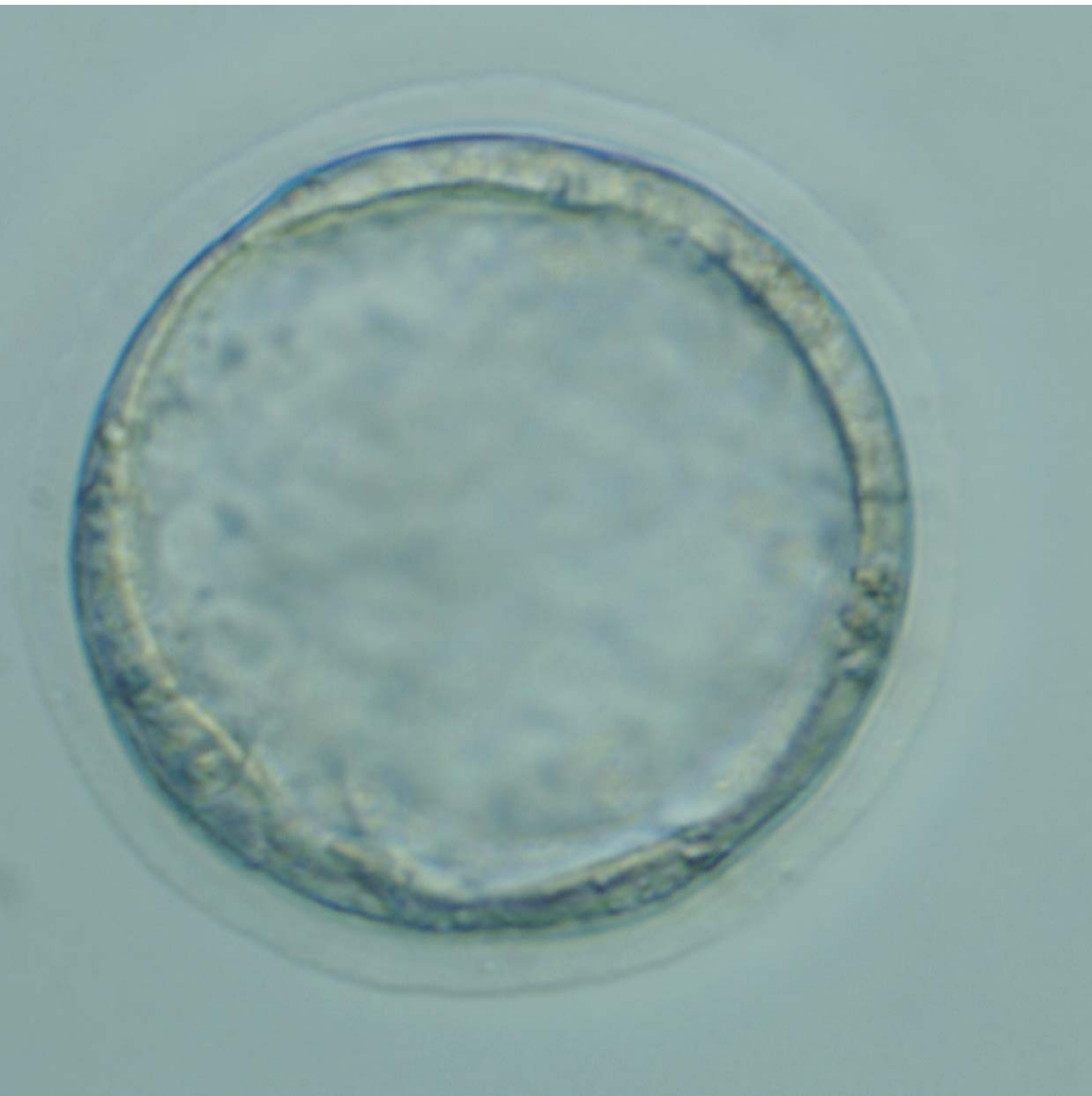

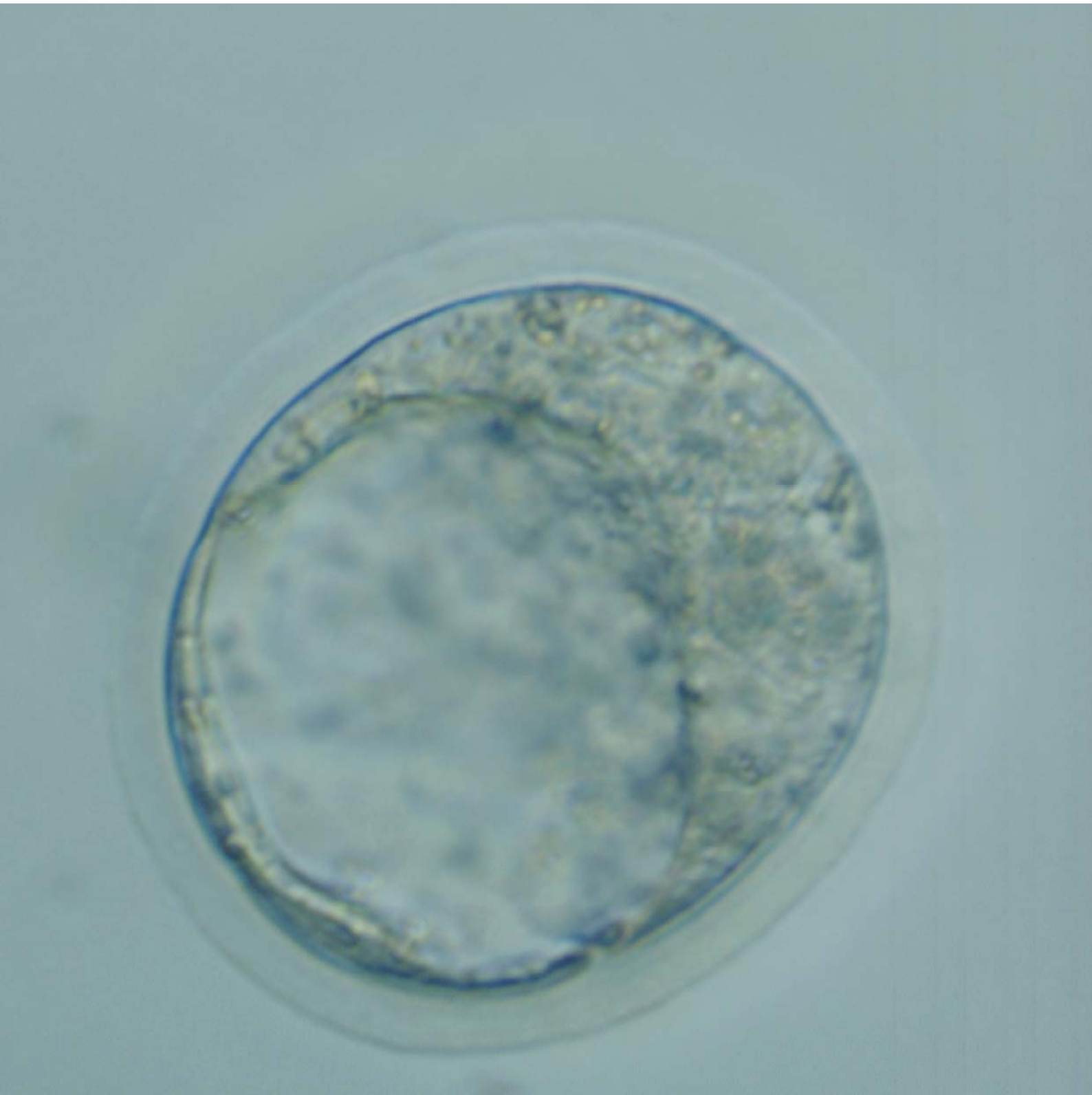

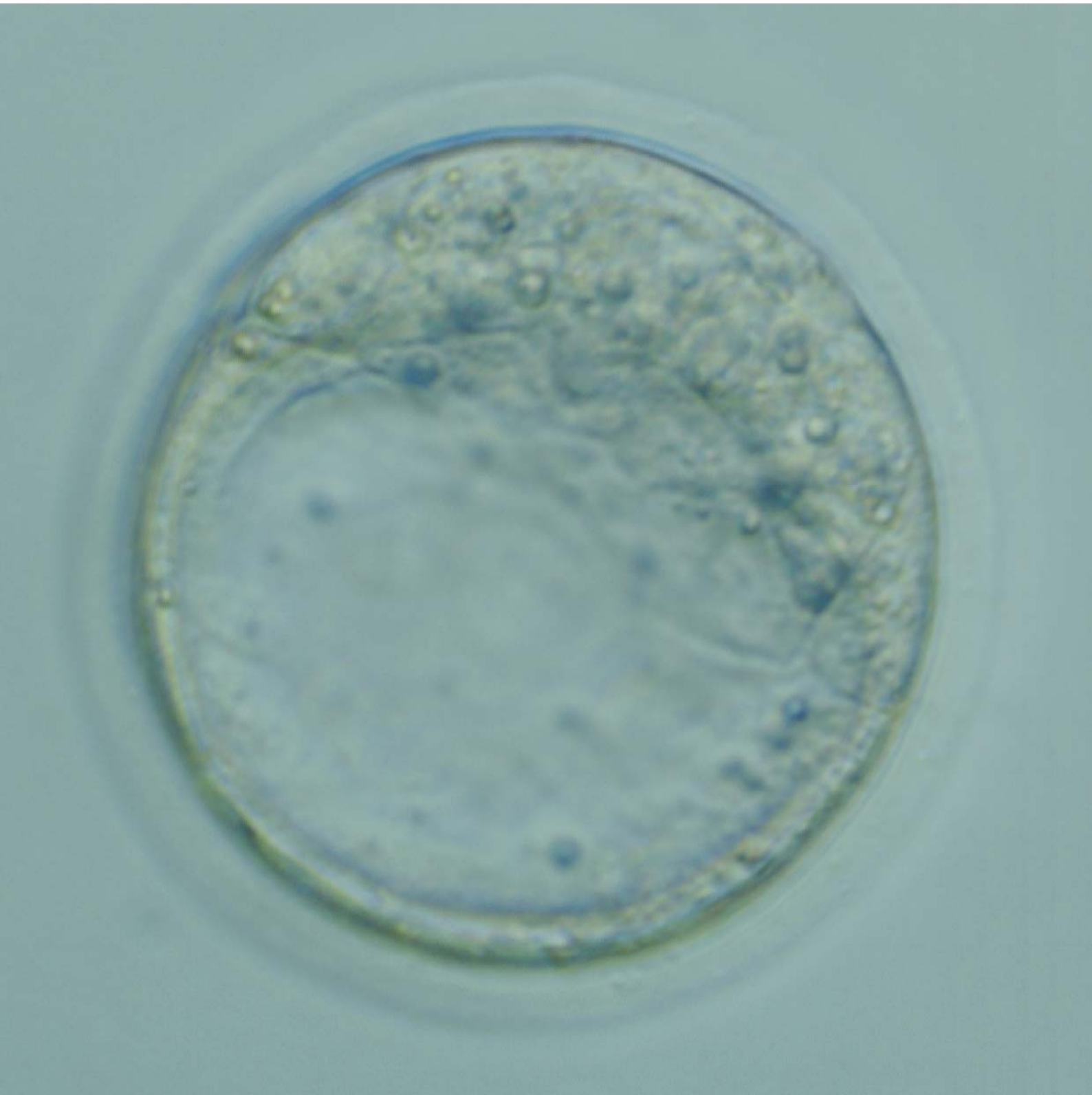

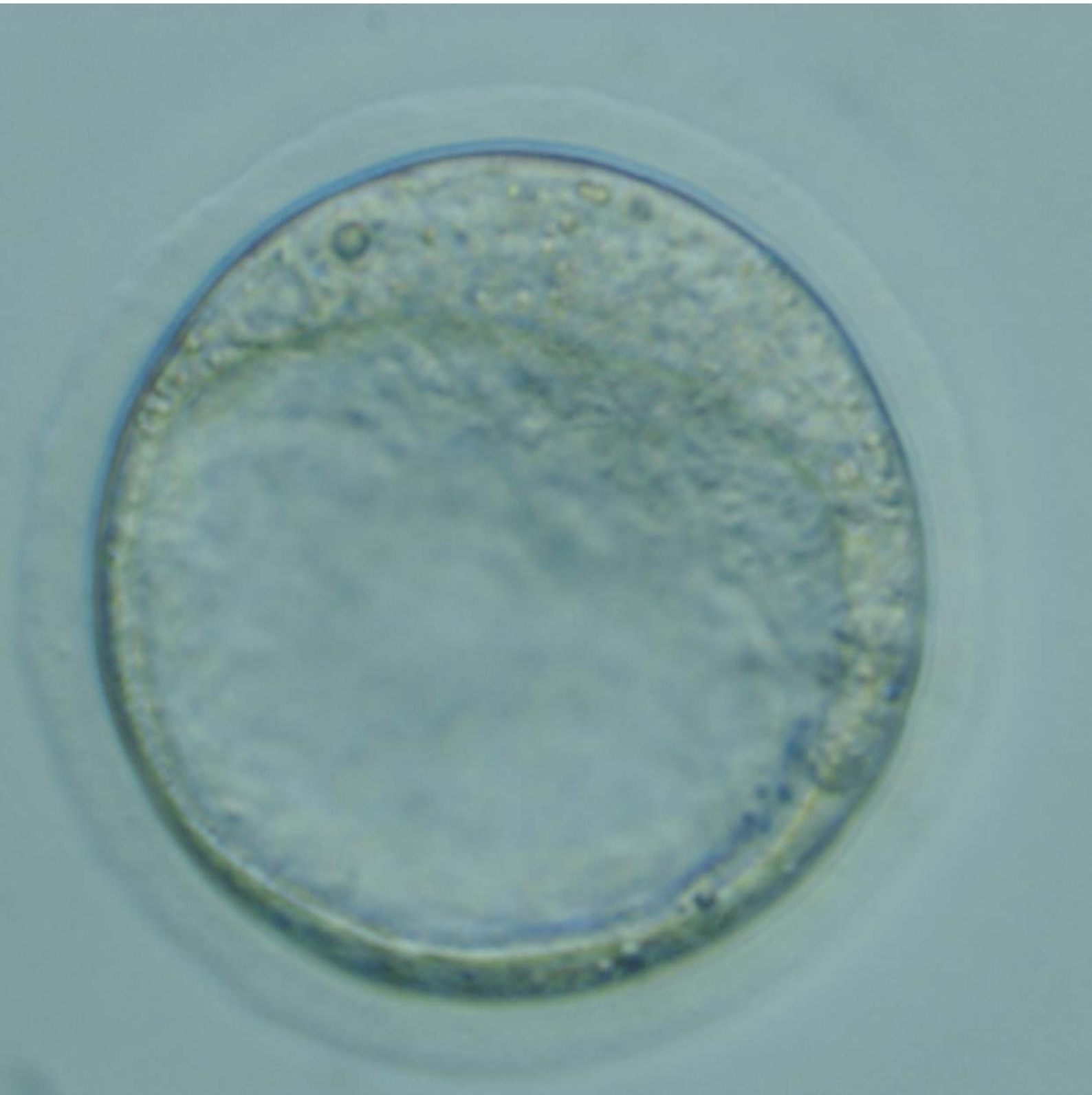

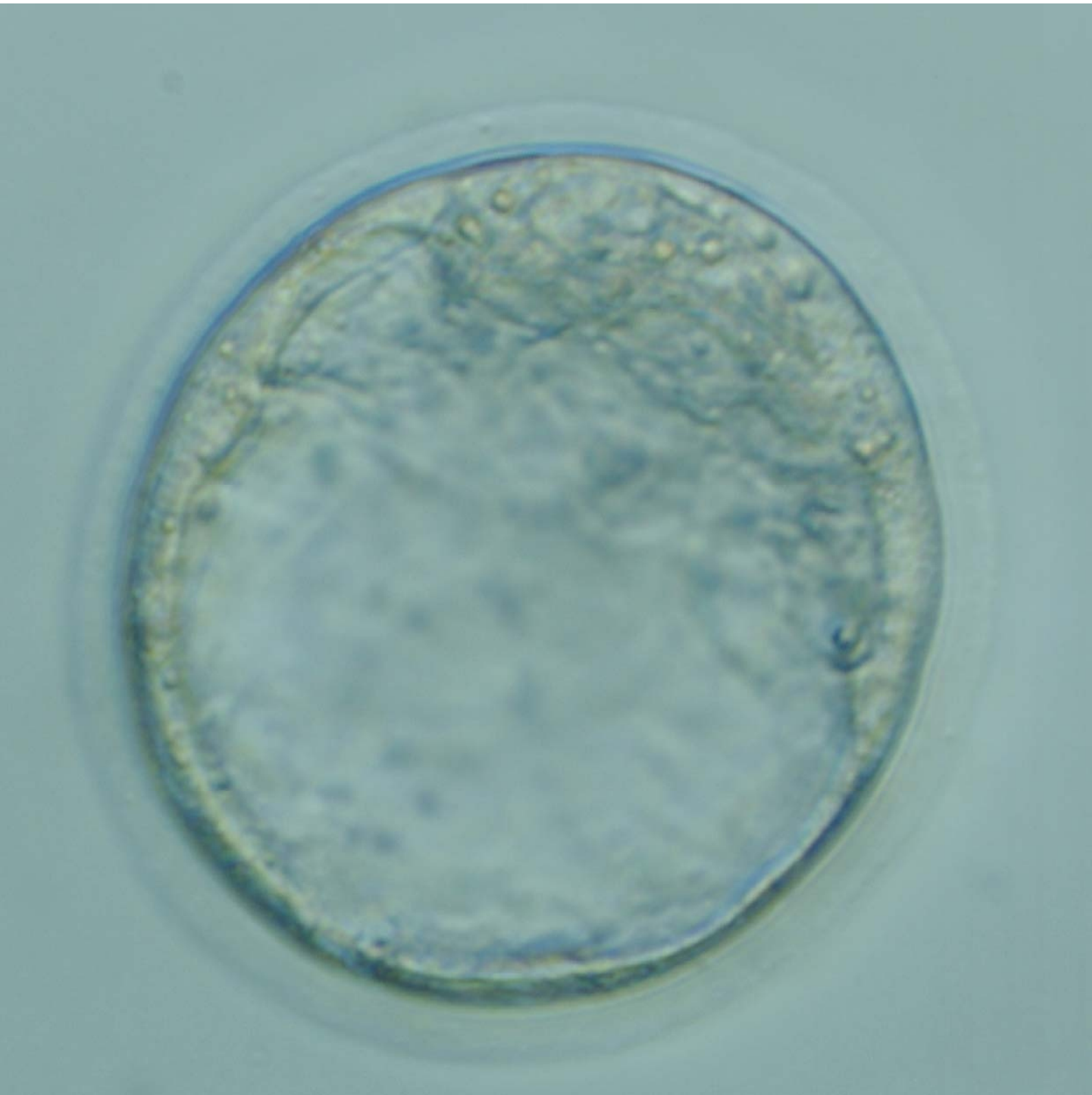

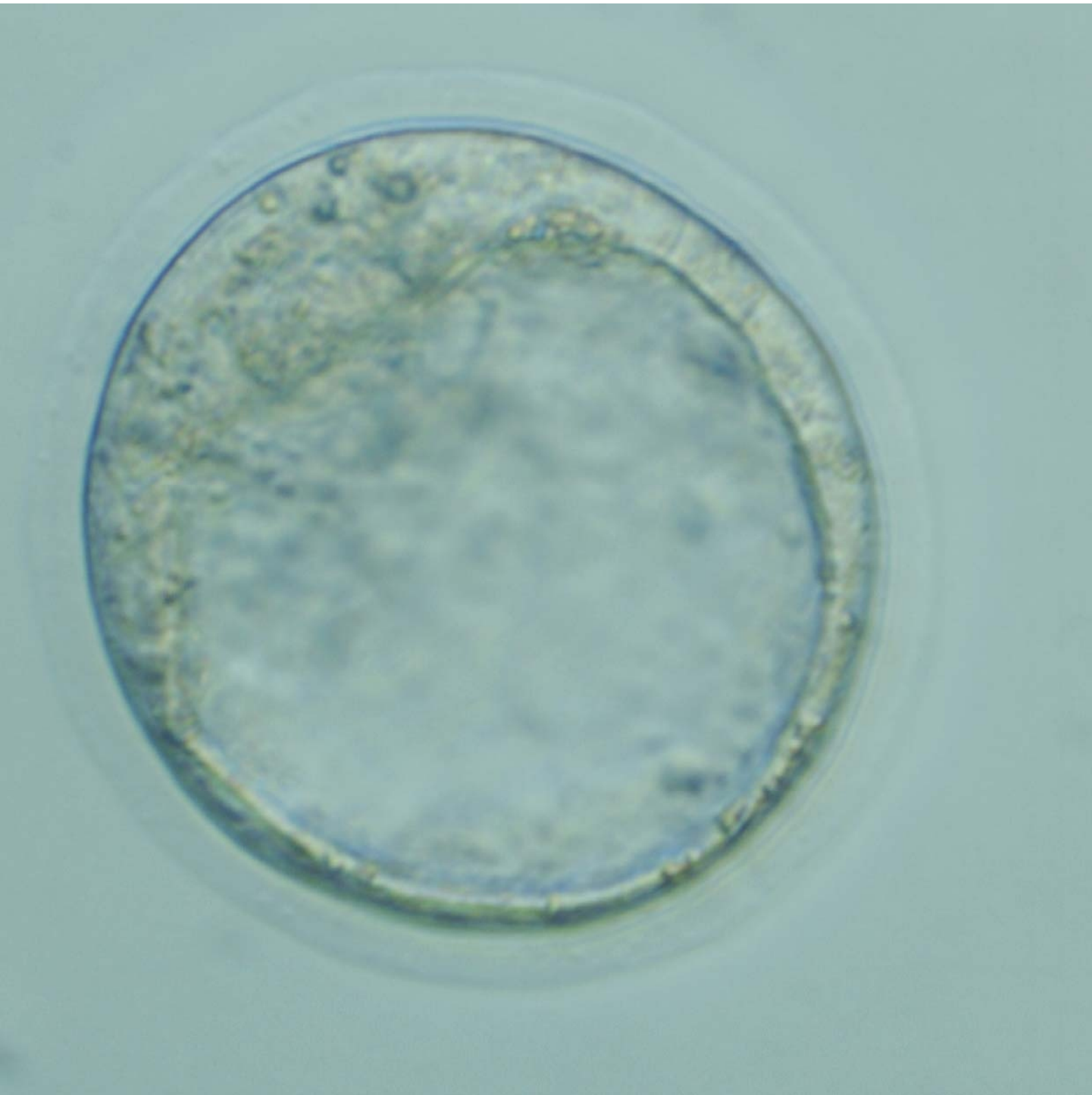

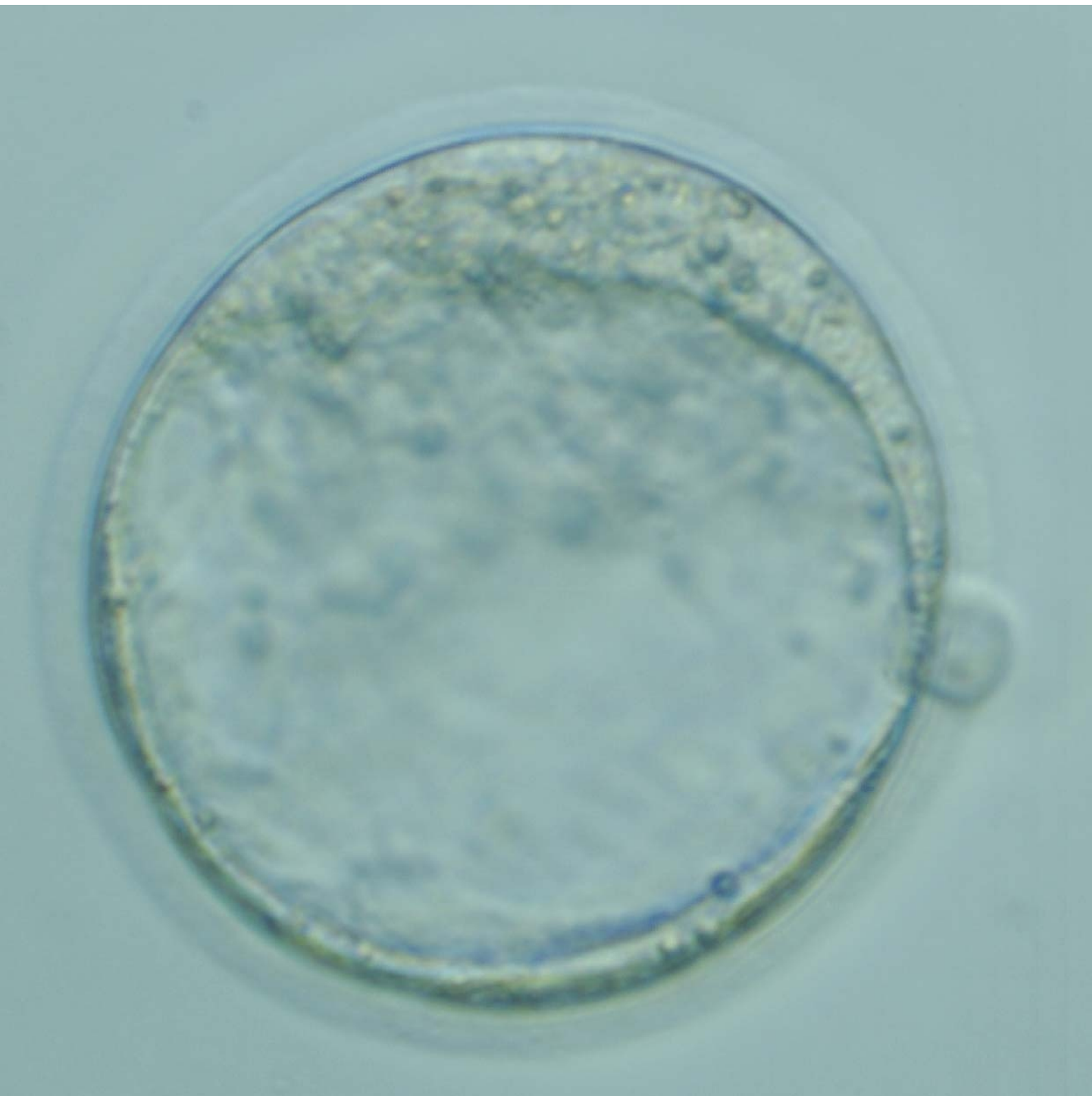

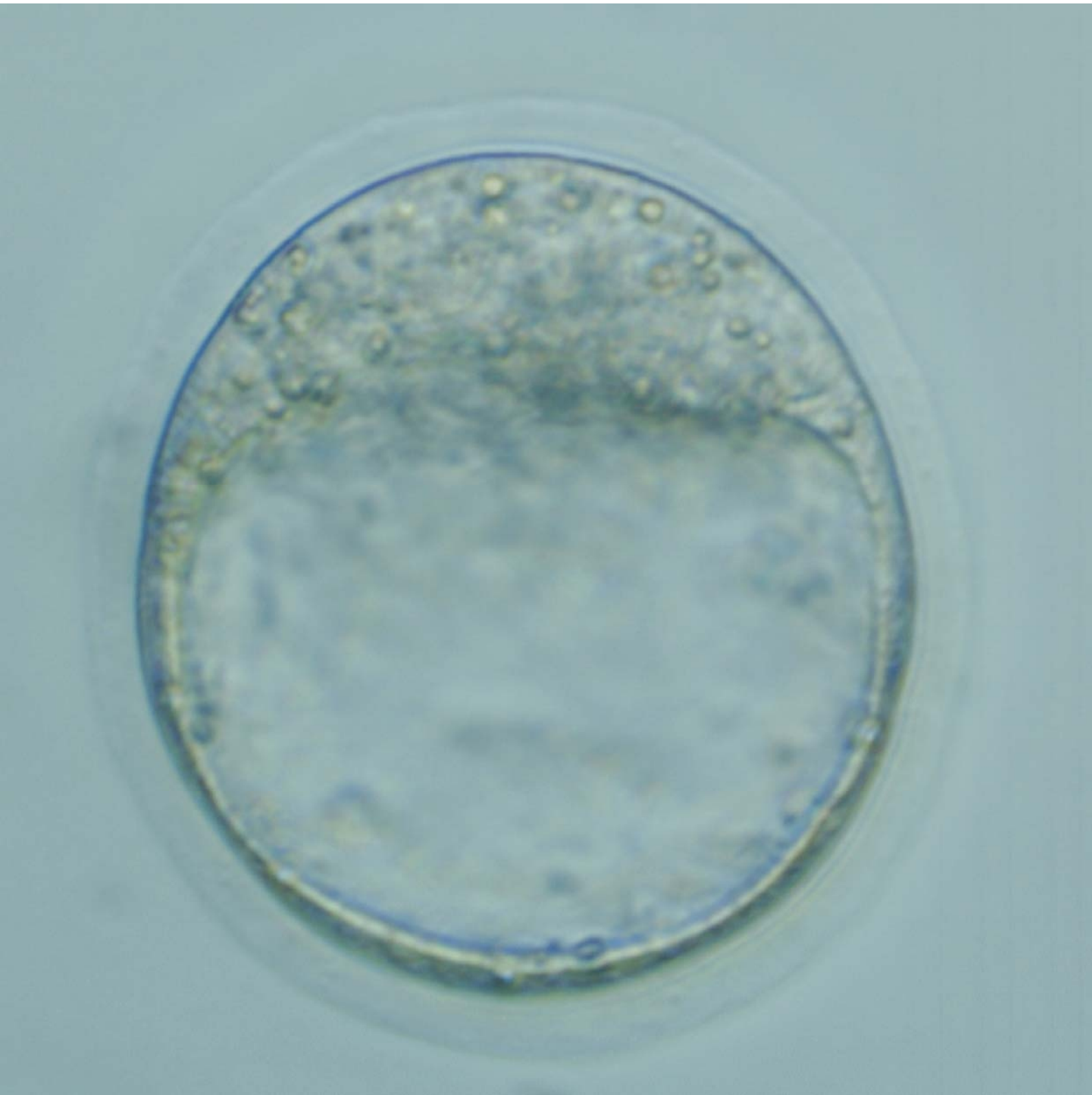

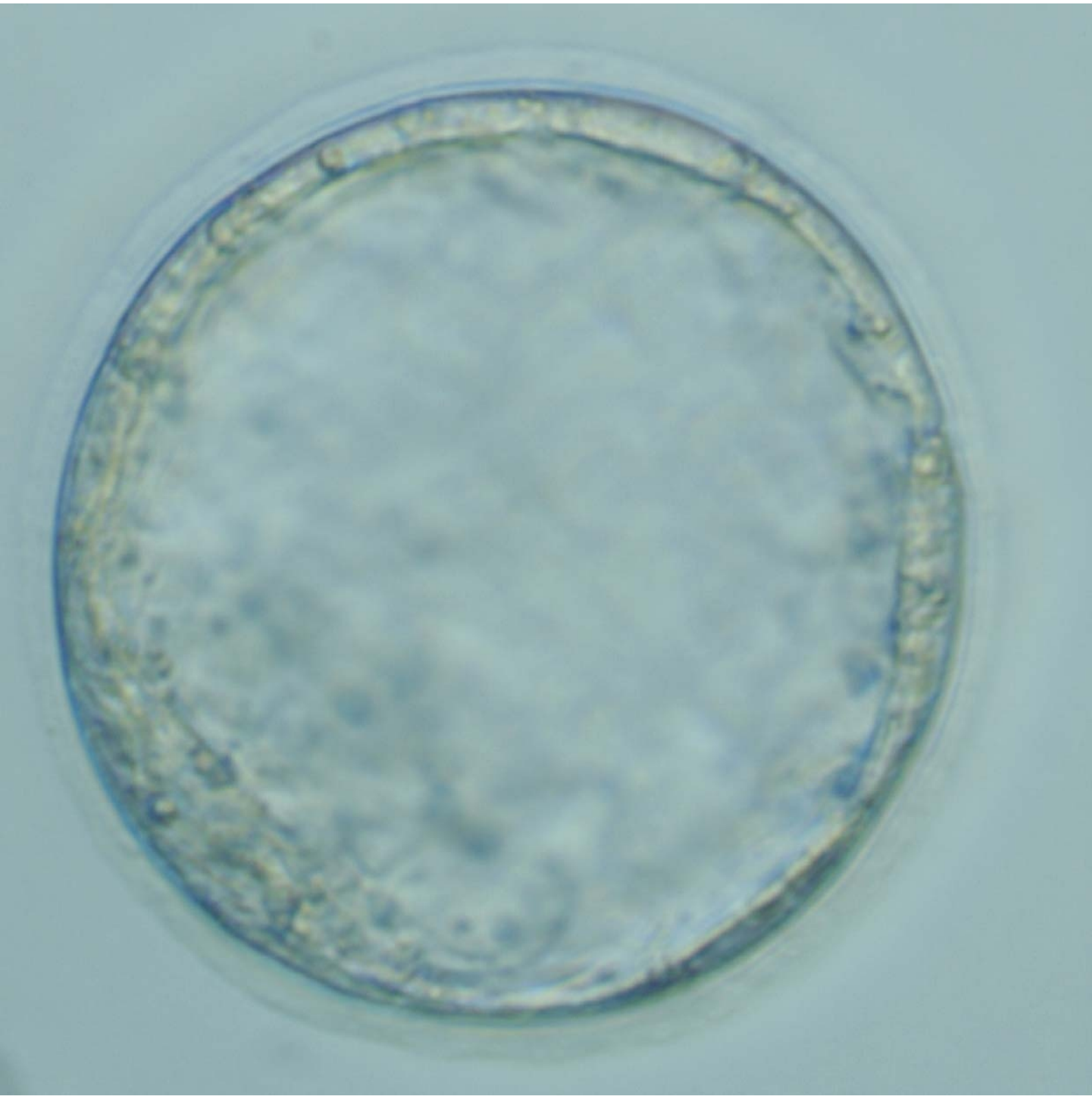

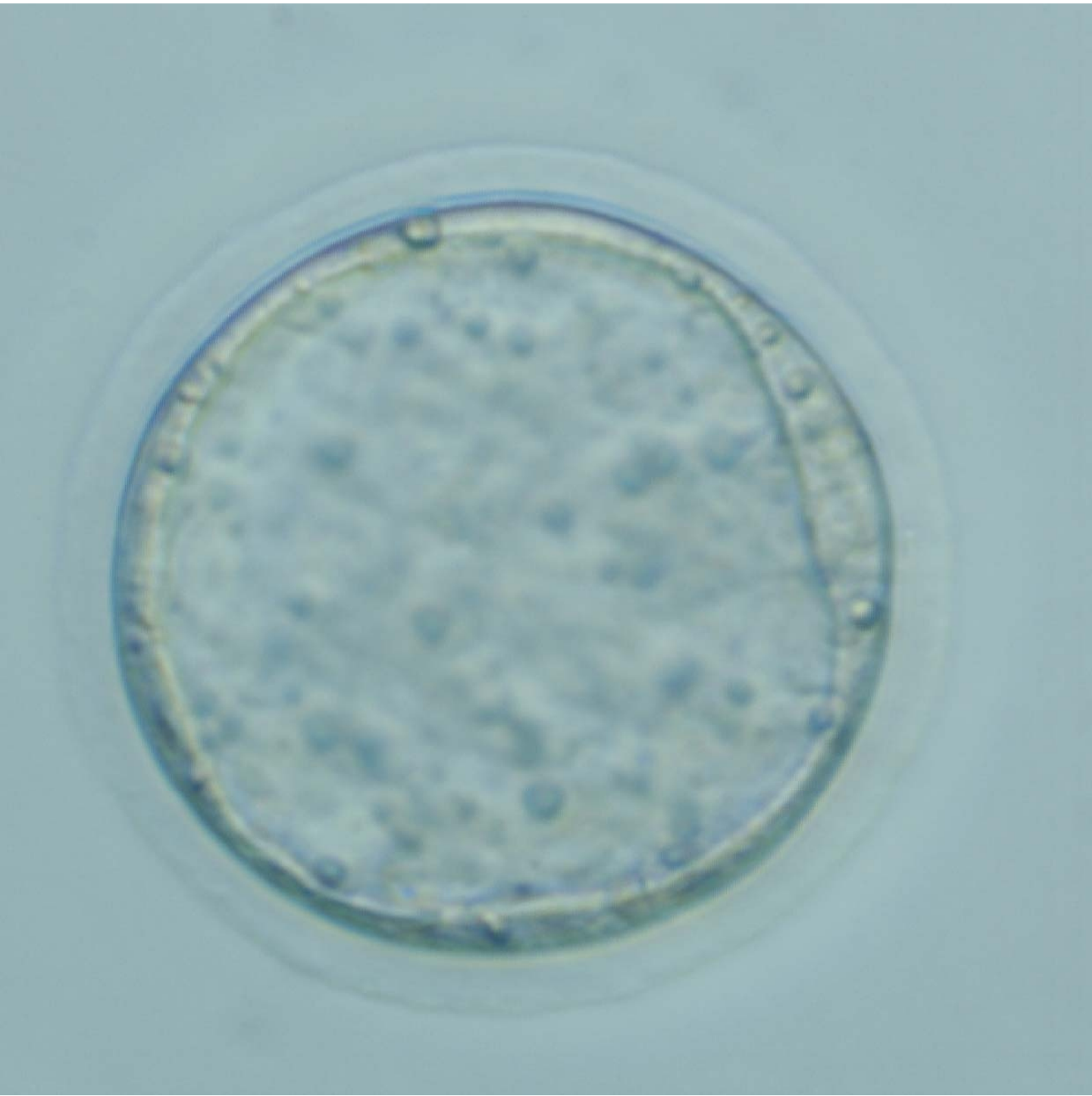

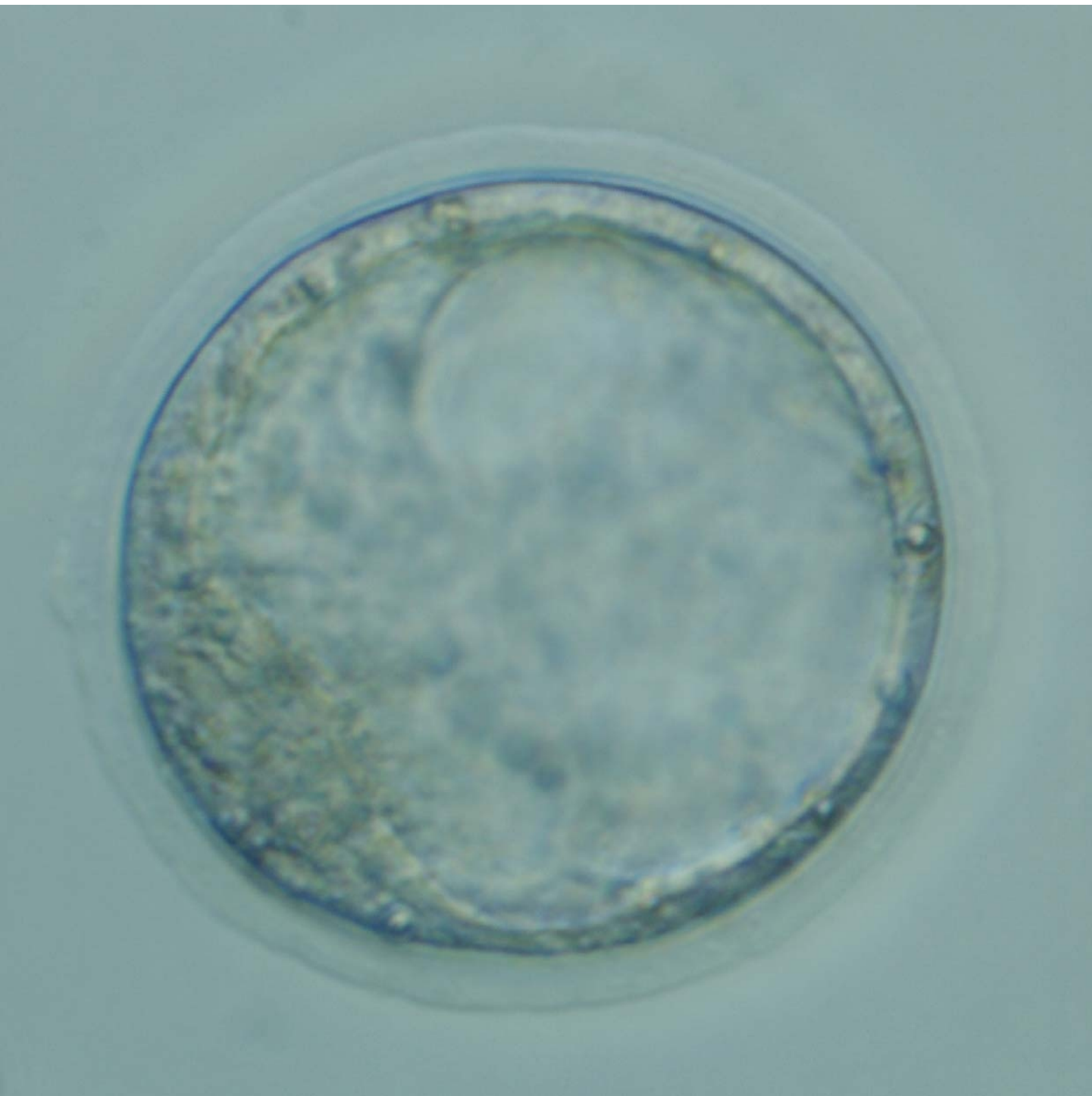

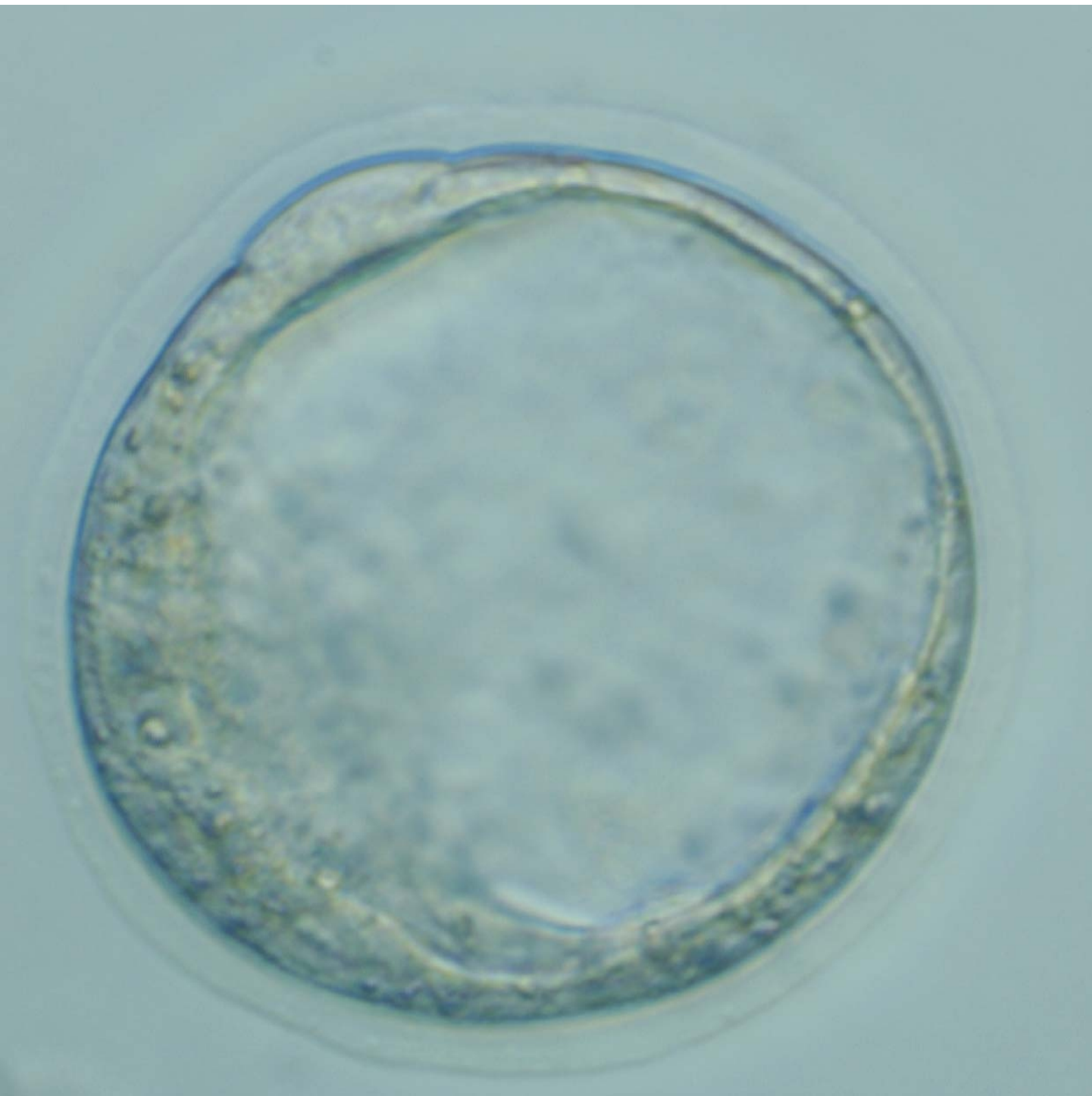

Supplement: Additional file 1 — Forty blastocysts. Forty photographs of individual blastocysts. After stringent procedures (mechanical decoronisation and washes), neither granulosa cells nor spermatozoa could be observed. [file 1471-213X-7-116-S1.pdf]
