# Supplementary material for: Assisted Reproductive Technology affects developmental kinetics, H19 Imprinting Control Region methylation and H19 gene expression in individual mouse embryos
Source: BMC Dev Biol. 2007 Oct 18;7:116. doi: 10.1186/1471-213X-7-116 (PMC2169233; doi:10.1186/1471-213X-7-116)
Supplement: Additional file 2 — Spermatozoa or granulosa cells observation. No contamination by sperm or granulosa cells could be missed since they are easily observed at the one cell stage. In fact since we consistently used stringent washing and decoronisation procedures, possible events of contamination could always be excluded (see additional file 1). [file 1471-213X-7-116-S2.pdf]

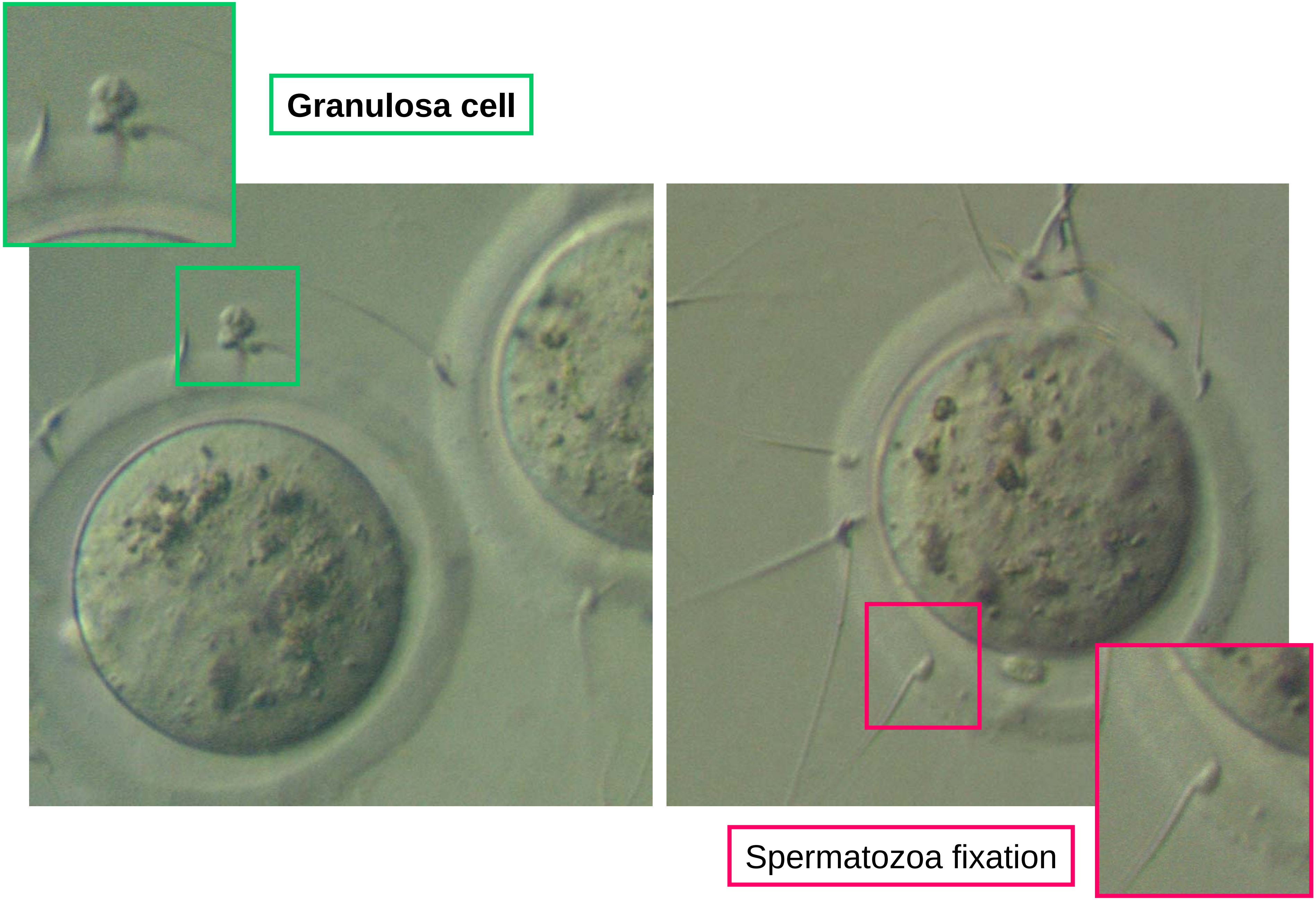
Granulosa cell
Spermatozoa fixation
